# Identification of a Diverse Core Set Panel of Rice From the East Coast Region of India Using SNP Markers

**DOI:** 10.3389/fgene.2021.726152

**Published:** 2021-11-25

**Authors:** Debjani Roy Choudhury, Ramesh Kumar, Vimala Devi S, Kuldeep Singh, N. K. Singh, Rakesh Singh

**Affiliations:** ^1^ Division of Genomic Resources, NBPGR, New Delhi, India; ^2^ Division of Germplasm Conservation, NBPGR, New Delhi, India; ^3^ NBPGR, New Delhi, India; ^4^ NIPB, New Delhi, India

**Keywords:** rice, SNP markers, genetic diversity, genotyping, SNP, coastal rice

## Abstract

In India, rice (*Oryza sativa* L.) is cultivated under a variety of climatic conditions. Due to the fragility of the coastal ecosystem, rice farming in these areas has lagged behind. Salinity coupled with floods has added to this trend. Hence, to prevent genetic erosion, conserving and characterizing the coastal rice, is the need of the hour. This work accessed the genetic variation and population structure among 2,242 rice accessions originating from India’s east coast comprising Andhra Pradesh, Orissa, and Tamil Nadu, using 36 SNP markers, and have generated a core set (247 accessions) as well as a mini-core set (30 accessions) of rice germplasm. All the 36 SNP loci were biallelic and 72 alleles found with average two alleles per locus. The genetic relatedness of the total collection was inferred using the un-rooted neighbor-joining tree, which grouped all the genotypes (2,242) into three major clusters. Two groups were obtained with a core set and three groups obtained with a mini core set. The mean PIC value of total collection was 0.24, and those of the core collection and mini core collection were 0.27 and 0.32, respectively. The mean heterozygosity and gene diversity of the overall collection were 0.07 and 0.29, respectively, and the core set and mini core set revealed 0.12 and 0.34, 0.20 and 0.40 values, respectively, representing 99% of distinctiveness in the core and mini core sets. Population structure analysis showed maximum population at K = 4 for total collection and core collection. Accessions were distributed according to their population structure confirmed by PCoA and AMOVA analysis. The identified small and diverse core set panel will be useful in allele mining for biotic and abiotic traits and managing the genetic diversity of the coastal rice collection. Validation of the 36-plex SNP assay was done by comparing the genetic diversity parameters across two different rice core collections, i.e., east coast and northeast rice collection. The same set of SNP markers was found very effective in deciphering diversity at different genetic parameters in both the collections; hence, these marker sets can be utilized for core development and diversity analysis studies.

## Introduction

Rice (*Oryza sativa* L.) is an important cereal crop which is a predominant food for over three billion people across the globe. Since it can adapt to an extensive spectrum of environmental conditions, it is therefore considered a varied crop species (Chang, 1976). In general, more genetically diverse crops will have an increased capacity to adapt with climatic conditions, while uniformity reduces the genetic diversity (Luan et al., 2006). There should be more significant variation in the breeding population so as to stimulate genetic gain from selection to improved yield, biotic and abiotic resistance to stress, and other traits. Genebanks with proper coordination can be explored to find genetic variations present in its accessions which can further help in finding advanced traits and selecting better parental combinations for developing lineage with maximum genetic variability (Barrett and Kidwell, 1998). Genetic diversity and population structure knowledge form the backbone in building core sets adequately representing variations found in the whole collection, and thereby making the collection small and condensed (Yan et al., 2007; Agrama et al., 2009; El Bakkali et al., 2013), thus creating standard data and calculating the potential loss of genetic diversity during conservation and management (Reif et al., 2005). Trait-specific germplasm identification from large collections is crucial to successful introduction of new diversity into the crop improvement programs (Upadhyaya et al., 2014). It is pivotal to comprehend the scope of genetic variation in crop germplasm and how to control it; results from molecular marker-based diversity studies should be used with caution, especially when it comes to germplasm conservation initiatives, the reason being adaptability, which plays a major role during the process of evolution and individuals’ survival in populations. As a result, it becomes ambiguous, determining whether plant selection is based on markers directly or on linked loci responsible for adaptive traits (Raybould et al., 1996).

Single-nucleotide polymorphisms (SNPs) are supposed to be the most bountiful variation found across the genome and therefore are excellent for high-resolution genotyping, in turn useful for analysis of genetic diversity and association mapping, linkage mapping, and marker-assisted selection (MAS) (Gonzaga et al., 2015). Simple sequence repeats (SSRs) are being replaced by SNPs at a very high degree. They have become a choice for utilization in plant breeding and genetics (McCouch et al., 2010). Using second-generation sequencing methods, scientists have been able to detect millions of SNPs in rice genome (Huang et al., 2009, 2010; Xu et al., 2011). Array-based SNP detection is currently one of the most popular high-throughput marker detection methods, allowing data to be evaluated in real time. Because of their abundance, locus specificity, low error rates, and co-dominant inheritance SNP assays are popular markers (Rafalski, 2002; Schlotterer, 2004). There are numerous multiplex and uniplex genotyping platforms are available (Syvänen, 2001; Chen and Sullivan, 2003). SNP genotyping platforms including Illumina BeadXpress (Yamamoto et al., 2010; Thomson et al., 2012), Affymetrix (Singh et al., 2015; McCouch et al., 2016), and the KASP marker system (Cheon et al., 2018; Yang et al., 2019) have been developed recently and applied to rice molecular breeding. In the year 2020, Seo et al. (2020) developed two 96-plex *indica-japonica* SNP genotyping sets using the Fluidigm platform; however, genotyping platforms are very expensive.

Plant Genetic Resources of native rice needs to be conserved promptly. A major conservation strategy counts on developing core collections, thereby screening gene bank accessions for important agronomic traits. To create a core collection, one needs a good construction strategy. Frankel (1984) and Brown (1989) suggested the concept of core collection; it is a small set of accessions from the entire collection with least amount of repetition and maximal genetic diversity of a species. It is usually 5% to 10% representation of the total population. Van Hintum et al. (2000), in the year 2000, outlined a comprehensive process for assembling a core collection. These can be summarized as 1) determine the overall sampling ratio; 2) partition them into groups; 3) decide on the percentage of the group that will be sampled; and 4) choose from each group entry. Several other strategies have been proposed to create a core collection such as PowerCore (Kim et al., 2007), MStrat (Gouesnard et al., 2001), stepwise clustering (Hu et al., 2000), and least distance stepwise clustering (Wang et al., 2007). These various methodologies are dependent on several parameters such as species’ genetic diversity, collection size, grouping, and data type (i.e., phenotypic or molecular data). However, if adequately characterized, a quality core collection can form a progressive collection for long-term conservation.

In the present study, we demonstrate the identification of a diverse core set panel from the east coast region of India comprising 247 core accessions and a mini core set of 30 accessions to enable elite gene mining for breeding and conservation and on a long-term perspective to form a panel for association studies. We have also done a comparative analysis of genetic diversity parameters across two different rice collections (northeast rice collection by Roy Choudhury et al., in 2014, and the coastal rice collection), thus validating the use of SNP markers for studying diversity parameters across any other rice collection.

## Materials and methods

### Germplasm resources

A set of 2,242 seed samples of the east coast region of India (Andhra Pradesh, Orissa, and Tamil Nadu) were procured from the Indian National Genebank, National Bureau of Plant Genetic Resources (NBPGR), New Delhi, with passport data (National ID, i.e., Indigenous Collection (IC) number) and the states to which they belong. This is given in Supplementary Table S1.

### Genomic DNA isolation and molecular characterization using SNP markers

The seed was de-husked, and genomic DNA was isolated using the QIAGEN DNeasy Plant Mini Kit. A tissue lyser (Retsch, Haan, Germany) and a tissue lyser adapter set (Qiagen, Hilden, Germany) was used to grind kernels into fine powder. Working stocks with 10 ng/μl of the genomic DNA samples was prepared, and 30 µl of the diluted sample was transferred to a 96-well plate to be run on the Sequenom MassARRAY which adopts the matrix-assisted laser desorption ionization-time of flight (MALDI-TOF) mass spectrometer for most authentic detection of SNPs (www.sequenom.com). The information about the genetic location of the multiform assays designed for 36 SNPs having conserved single-copy rice genes was derived from Singh et al. (2007). Sequenom Corporation (San Diego, CA, USA) created and validated a 36-plex assay with three genes per chromosome. The assay Design 3.1 program was used to build pre-amplification primers and genotyping primers, purchased and utilized for SNP validation according to the Sequenom user manual’s methodology. There were two polymerase chain reactions (PCR). The first was a normal PCR of 45 cycles with pre-amplification primers followed by removal of unincorporated dNTPs. After adjusting genotyping primers, the second PCR was an extension PCR. The extension rate was enhanced by increasing the number of cycles to 300, thus giving the highest call rates. Call rates and extension rates were also adjusted according to genotype calling algorithms. MassARRAY Typer Analyzer 3.4 Software was used to visualize SNP calling.

### Genetic diversity and phylogenetic analyses

The results of SNP data were subjected to analysis using PowerMarker (V3.25) (Liu and Muse, 2005) to calculate major allele frequency, heterozygosity, gene diversity, and PIC (polymorphic information content). The genotypes’ genetic distances (Nei et al., 1983) were computed, and a neighbor joining (NJ) tree was generated and viewed in FigTree v 1.4.3 (Rambaut, 2010). To infer historical origin, software STRUCTURE V2.3.1 was used which provides clusters of related genotypes (Pritchard et al., 2000). To infer the value of genetic cluster (K), each sample was run from K = 2 to K = 10 with the admixture model and correlated allele frequency. Each K run was replicated thrice with 100,000 burn-in period and 100,000 Monte Carlo Markov Chain replicates (Evanno et al., 2005). The analysis was carried out regardless of the accessions’ geographical origin. The dataset optimal K value was obtained using program “structure harvester” (http://taylor0.biology.ucla.edu). Additional hierarchical structure analysis was done after observing additional peaks at two different K values to unmask the groups (Ambreen et al., 2018). Accessions with membership proportions >80% were considered as pure, whereas membership proportions less than 80% were judged as admixed. The software GenAlEx V6.5 (Peakall and Smouse, 2012) was used to perform principal coordinate analysis (PCoA) and analysis of molecular variance (AMOVA) between the STRUCTURE populations.

### Development of core collection

PowerCore 1.0 software (Kim et al., 2007) was used to develop the core. The data set was in simple excel format, and the first column in the data set was given the name % Accessions and the subsequent columns were named NM1, NM2, and so on. After loading the data set in the software, settings were set to heuristic search with maximum possible entries. The diversity index was also mentioned in the same window of the software. There were a number of runs carried out to extract the best possible entries in the core set. Since the number of rice accessions from each state differed ranging from 1,133 accessions from Andhra Pradesh, 378 accessions from Orissa, and 731 accessions from Tamil Nadu, therefore, to avoid individual state collections taking precedence over the core, a separate core subset was developed using the rice collections from each state. Each state accession was analyzed for maximum genetic diversity parameters forming the basis of core subsets. Finally, the core accessions from all the three states were gathered together to develop a core of coastal Indian rice of 247 accessions (126 accessions from Andhra Pradesh, 45 accessions from Orissa, and 76 accessions from Tamil Nadu) (Supplementary Table S2). A mini core comprising 30 accessions was also generated using PowerCore (Supplementary Table S3). This core collection is of core collection type I (CC-I) giving a uniform representation of the original population. Here, each entry in the core set has one or more accessions that jointly make up the whole collection (Odong et al., 2013). Shannon’s diversity index and Nei’s gene diversity index were used to evaluate the diversity captured in the core and mini core collection relative to the entire collection using PowerCore. The statistical analysis of the core set and the mini core set was done using PowerMarker (V3.25) (Liu and Muse, 2005) to calculate major allele frequency, gene diversity, heterozygosity, and PIC. This has been done to give a complete depiction of diversification in the total collection and string out the best core set which can stand out as a benchmark collection.

### Kinship analysis of the core collection

Kinship analysis was used to determine shared ancestry between individuals in the core collection. Tassel v 5 (Bradbury et al., 2007) was used to create a kinship matrix. All the negative values were considered as zero, and a simple bar graph was prepared using Microsoft Excel. An interactive heat map was prepared using the online available tool https://build.ngchm.net/NGCHM-web-builder, NG-CHM BUILDER: Interactive Heat Map (Ryan et al., 2020).

### Validation of 36-Plex SNP assay in east coast rice collection and northeast rice collection

Genetic validation of 36-plex SNP assay (Table1) was done by comparing the genetic parameters (e.g., PIC, gene diversity, major allele frequency, and heterozygosity) in northeast rice (Roy Choudhury et al. in 2014) and east coast rice collection. A comparative analysis of these parameters across the two populations was summarized.

**TABLE 1:** List of SNP primers used for genotyping of 2,242 rice accessions along with gene diversity, heterozygosity, PIC, and major allele frequency.

Chromosome no	Marker name	Physical	Amplification primer1	Amplification primer2	GeneDiversity	Heterozygosity	PIC	Major.Allele.Frquency
1	01-3916-1_C	25381654	ACG​TTG​GAT​GGG​GTT​TGC​ATG​TTA​ATA​GGG	ACG​TTG​GAT​GCC​GAA​TCT​CTA​TCA​AGG​AAG	0.4700	0.0450	0.3596	0.6224
	01-608-4_C	3421011	ACG​TTG​GAT​GAG​GAC​CAT​CTT​CTT​GCA​CTG	ACG​TTG​GAT​GCC​ATT​TGC​AAG​GCC​CAT​TTC	0.4981	0.1173	0.3740	0.5312
	01-6351-1_C	40914292	ACG​TTG​GAT​GGT​TGG​AAC​ACA​TGA​TTT​CAC	ACG​TTG​GAT​GAT​CTC​TTT​GGA​CAG​AGT​CCC	0.2298	0.0358	0.2034	0.8676
2	02-267_C	1570149	ACG​TTG​GAT​GGT​CAA​TCT​TGC​AGG​AGT​TGG	ACG​TTG​GAT​GTG​GCT​CCT​CTT​CTC​CGG​TCT	0.4060	0.0972	0.3236	0.7168
	02-3029-1_C	18821156	ACG​TTG​GAT​GTG​TCT​GCA​ATA​ACT​TGT​GCC	ACG​TTG​GAT​GAA​ATC​AGC​TGC​AGC​ATT​ACC	0.4096	0.0864	0.3257	0.7126
	02-4333-1_C	28688819	ACG​TTG​GAT​GGG​AAT​GTT​TAG​TTT​TGA​GG	ACG​TTG​GAT​GTG​TAG​GTG​CTA​CTT​GCT​TCC	0.3282	0.0494	0.2743	0.7931
3	03-1691-1_C	10849512	ACG​TTG​GAT​GAA​CAA​CGC​CAG​GAA​CAT​CAC	ACG​TTG​GAT​GAA​GCG​GCT​CAA​GGT​ACA​ATC	0.3208	0.0389	0.2693	0.7993
	03-3478-1_C	22815422	ACG​TTG​GAT​GCC​TGC​AGC​AAA​CGC​CAA​TTT	ACG​TTG​GAT​GTC​AGG​TAA​CCG​ATC​GAT​TTG	0.4964	0.1558	0.3732	0.5425
	03-4660-1_C	31020366	ACG​TTG​GAT​GCT​CCC​ATC​CTA​GTA​TCC​ATC	ACG​TTG​GAT​GTG​CCT​TCT​CTT​ACA​GGT​TCC	0.3987	0.0816	0.3192	0.7251
4	04-1801-20_C	11859836	ACG​TTG​GAT​GCC​CTC​AAA​AAA​AAG​TTG​TAA​G	ACG​TTG​GAT​GCA​GTA​AAT​TTC​CAG​GGA​GAT​A	0.4208	0.4270	0.3323	0.6990
	04-19-4_C	225838	ACG​TTG​GAT​GTC​TAC​ACA​TTA​GCT​CGC​TGG	ACG​TTG​GAT​GAC​AGT​AAC​CAC​AAT​ATG​CCG	0.0198	0.0100	0.0196	0.9900
	04-3787-3_C	25211800	ACG​TTG​GAT​GTT​ATC​TCT​GCT​TGC​TCG​CTC	ACG​TTG​GAT​GAA​GTA​TCT​GCC​CCA​AGT​GAC	0.4387	0.0914	0.3425	0.6750
5	05-2692-1_C	18783426	ACG​TTG​GAT​GGA​ACT​TTA​CTC​TCA​GTA​CA	ACG​TTG​GAT​GTG​GTT​TGA​TGA​GTC​GTT​TGC	0.1981	0.0388	0.1785	0.8885
	05-4192-1_C	28065769	ACG​TTG​GAT​GAG​TTT​GTT​GAC​AGC​AGA​ACC	ACG​TTG​GAT​GTA​GCT​TAC​TAG​TTC​ATG​TG	0.4947	0.1062	0.3723	0.5515
	05-48-1_C	287362	ACG​TTG​GAT​GCA​GAG​ATG​TCT​GTT​GTT​AGC	ACG​TTG​GAT​GCA​ACC​AGG​GAT​ACA​ATA​TGA​C	0.4584	0.1251	0.3533	0.6443
6	06-1256-1_C	7573979	ACG​TTG​GAT​GCA​CGT​GCC​TAT​GAT​TAG​CAG	ACG​TTG​GAT​GGA​TCG​TTT​ACT​TCT​TTG​CCC	0.0593	0.0112	0.0576	0.9694
	06-1776-1_C	11093772	ACG​TTG​GAT​GGG​GCC​AAT​TTG​CTT​AGT​GC	ACG​TTG​GAT​GAG​CAT​AAG​GTA​TTA​AAG​TC	0.2320	0.0698	0.2051	0.8660
	06-2509-1_C	15737387	ACG​TTG​GAT​GCC​TTC​GCG​CTT​GCA​ATT​TGG	ACG​TTG​GAT​GAA​ATC​AGC​ACG​CGT​CAA​CAC	0.2046	0.0303	0.1837	0.8843
7	07-2904-39_C	19160255	ACG​TTG​GAT​GAA​TGG​TGG​TGT​ATC​TTG​AGC	ACG​TTG​GAT​GGG​TGT​GAC​TTC​TCA​TGA​CAG	0.2541	0.0569	0.2218	0.8506
	07-293-12_C	1859603	ACG​TTG​GAT​GCA​CTA​ATT​CTT​GGT​ATT​ATG​G	ACG​TTG​GAT​GTC​AAT​GTG​TTC​TCA​CAG​ACC	0.1173	0.0260	0.1105	0.9374
	07-4304_C	2782410	ACG​TTG​GAT​GCA​CGT​GCC​TAT​GAT​TAG​CA	ACG​TTG​GAT​GGA​TCG​TTT​ACT​TCT​TTG​CC	0.4635	0.0592	0.3561	0.6351
8	08-2765-2_C	18084851	ACG​TTG​GAT​GTC​CCT​CCA​TGT​TGT​GAG​TTC	ACG​TTG​GAT​GCT​TGC​AAG​AGA​CAT​CCA​AGA	0.1636	0.0175	0.1502	0.9101
	08-4218-5_C	27692470	ACG​TTG​GAT​GGG​TGG​ACA​AAG​ATA​AGG​AAG	ACG​TTG​GAT​GGA​CTG​GAA​ATA​TAC​TCC​CTC	0.4658	0.1166	0.3573	0.6307
	08-847-6_C	5399913	ACG​TTG​GAT​GCC​CAA​CGT​ATT​AAT​GGC​AAC	ACG​TTG​GAT​GGC​TGT​GTA​GTA​ATT​TGC​CTG	0.4754	0.1366	0.3624	0.6109
9	09-209_C	1297966	ACG​TTG​GAT​GGA​GGC​AAA​AGG​CAA​ACC​GAC	ACG​TTG​GAT​GGA​CTT​GAG​CGA​GTC​GAT​GTC	0.2144	0.0419	0.1914	0.8779
	09-2107-5_C	13705487	ACG​TTG​GAT​GTG​ACC​ACA​CCA​CAC​AAA​CAC	ACG​TTG​GAT​GGG​GAT​TTG​CGG​TTT​TTG​GAC	0.2831	0.0627	0.2430	0.8294
	09-2716-4_C	19541336	ACG​TTG​GAT​GTG​AGC​CAC​AGA​TTC​CCT​TTC	ACG​TTG​GAT​GCT​CGA​GTA​ATT​CAA​AAC​CAC	0.2056	0.0556	0.1845	0.8836
10	10-1192-7_C	8122635	ACG​TTG​GAT​GCT​TTG​CTA​CGG​ATA​AAA​TG	ACG​TTG​GAT​GTC​ATG​CAA​ATA​CAG​ACA​TGG	0.4980	0.1228	0.3740	0.5318
	10-188-1_C	1218215	ACG​TTG​GAT​GGC​GCC​AGT​GTA​TGG​AAA​AAG	ACG​TTG​GAT​GGT​CCA​TAA​CAT​CAT​GGA​CTC	0.2492	0.0940	0.2182	0.8541
	10-2723_C	20696970	ACG​TTG​GAT​GCC​CAC​AAT​GAG​ATG​CAG​ATG	ACG​TTG​GAT​GAG​ACA​AAA​TGC​AAC​ACT​CCG	0.0754	0.0538	0.0725	0.9608
11	11-1849_C	11974790	ACG​TTG​GAT​GCG​CCA​CTC​TTC​CTG​ATT​TAG	ACG​TTG​GAT​GAC​AGA​TAC​GGG​AGG​CAT​TTC	0.1747	0.0487	0.1594	0.9033
	11-3935_C	28434679	ACG​TTG​GAT​GAT​CCC​TGA​GAC​TTT​GGA​TGG	ACG​TTG​GAT​GCC​AAC​TTG​AAT​GTC​CAT​TCC	0.1239	0.0273	0.1162	0.9337
	11-522-1_C	3033366	ACG​TTG​GAT​GCT​ACA​TGG​TAT​CAG​ATA​CCG	ACG​TTG​GAT​GAG​AAG​CGA​ACG​CGG​AAA​AAG	0.4596	0.0971	0.3540	0.6421
	12-1794_C	11215946	ACG​TTG​GAT​GGT​GAG​CCC​CAA​AAG​TTG​GTG	ACG​TTG​GAT​GTA​AGG​TCC​AGT​TTG​CTT​GGT	0.0287	0.0094	0.0283	0.9855
12	12-3200-2_C	21396181	ACG​TTG​GAT​GGC​TCA​AAC​CTA​GCA​ATA​ACT​G	ACG​TTG​GAT​GCC​TCC​TTC​CTA​CAA​GTT​TAA	0.0974	0.0314	0.0927	0.9487
	12-400_C	2160546	ACG​TTG​GAT​GCC​AAT​AGA​GTC​CAT​CTC​AGC	ACG​TTG​GAT​GGC​ACG​AGG​ATT​TAA​GAC​AGC	0.2585	0.0990	0.2251	0.8475
	Mean				0.2970	0.0770	0.2412	0.7848

## Results

### Genetic diversity of the total rice collection

Genotyping of the 2,242 rice accessions were performed using 36 SNP markers. The markers generated 72 alleles with a mean of two alleles per locus for the entire 2,242 coastal collection (Table 1). The maximum PIC was 0.37 for markers 01-608-4_C and 03-3478-1_C, and the minimum was 0.01 for marker 04-19-4_C with an average value of 0.24. The maximum and minimum heterozygosity was 0.42 for marker 04-1801-20_C and 0.009 for marker 12-1794_C, respectively, with a mean value of 0.07. Likewise, maximum and minimum gene diversity was found to be 0.49 for marker 01-608-4_C and 0.01 for marker 04-19-4_C, respectively, with an average of 0.29. The maximum major allele frequency was 0.99 for marker 04-19-4_C, and the minimum major allele frequency was observed to be 0.53 for marker 01-608-4_C with a mean value of 0.78 (Table 1).

### Phylogenetic analysis of the total rice collection

Cluster analysis of 2,242 rice accessions was performed and PowerMarker (v3.25) was used to determine the dissimilarity matrix which was used to construct an unrooted phylogenetic tree using FigTree v1.4.3 (Supplementary Figure S1). Total collection got grouped into three major groups. However, no differentiation could be made between these groups based on their geographical origin with each group displaying a heterogeneous clustering of individuals.

### Genetic diversity of the rice collection of the three coastal states (Andhra Pradesh, Orissa, and Tamil Nadu)

The rice collections of coastal states belonging to the east coast of India were analyzed for the genetic diversity and population structure study. The lowest PIC recorded was 0.0 for marker 04-19-4_C from the state of Orissa, and the highest was 0.37 for marker v03-3478-1_C from the state of Andhra Pradesh. Heterozygosity was observed to be 0.0 for three markers 04-19-4_C, 11-3935_C, and 12-3200-2_C from Orissa state, and the highest value observed was 0.69 for marker 04-1801-20_C from the state of Andhra Pradesh. The lowest value of genetic diversity was found to be 0.0 for marker 04-19-4_C, and the highest was 0.49 for marker 02-267_C both from Orissa. The lowest and highest major allele frequencies recorded were 0.52 for 02-267_C and 1.0 for 04-19-4_C, respectively, from Orissa state (Table 2). For the collections from three states, phylogenetic analysis was done using the neighbor joining (NJ) method, and an unrooted NJ tree was created. Rice collection from the state of Andhra Pradesh got clustered into two major groups. Group 1 had 220 accessions, and group 2 had 913 accessions (Supplementary Figure S2). There were two major groups each in case of rice collection from Orissa (Supplementary Figure S3) and Tamil Nadu (Supplementary Figure S4). The NJ tree of rice collection of Orissa exhibited 7 accessions in group 1, and 371 accessions got clustered in group 2. The NJ tree of rice collection of Tamil Nadu exhibited 5 accessions in group 1 and 726 accessions in group 2.

**TABLE 2:** List of genetic diversity parameters estimated for three east coast states, core, and mini core set.

	Sample size	Major.Allele.Frquency	Gene diversity	Heterozygosity	PIC
Andhra Pradesh	1133	(0.55–0.98) 0.78	(0.02–0.49) 0.30	(0.008–0.68) 0.09	(0.02–0.37) 0.25
Andhra Pradesh Core	126	(0.52–0.92) 0.74	(0.13–0.49) 0.36	(0.04–0.69) 0.15	(0.12–0.37) 0.29
Orissa	378	(0.52–1.0) 0.80	(0.00–0.49) 0.26	(0–0.12) 0.05	(0–0.37) 0.21
Orissa core	45	(0.5–1.0) 0.77	(0.0–0.5) −0.32	(0.0–0.18) 0.08	(0.0–0.37) 0.26
Tamil Nadu	731	(0.55–0.98) 0.82	(0.02–0.49) 0.24	(0.008–0.68) 0.07	(0.02–0.37) 0.20
Tamil Nadu core	76	(0.50–0.97) 0.79	(0.05–0.49) 0.29	(0.020–0.33) 0.10	(0.05–0.37) 0.23
core	247	0.7547	0.3417	0.1231	0.2759
Mini core	30	0.68	0.4	0.2	0.32

### Population structure of the total rice collection

**FIGURE 1:**
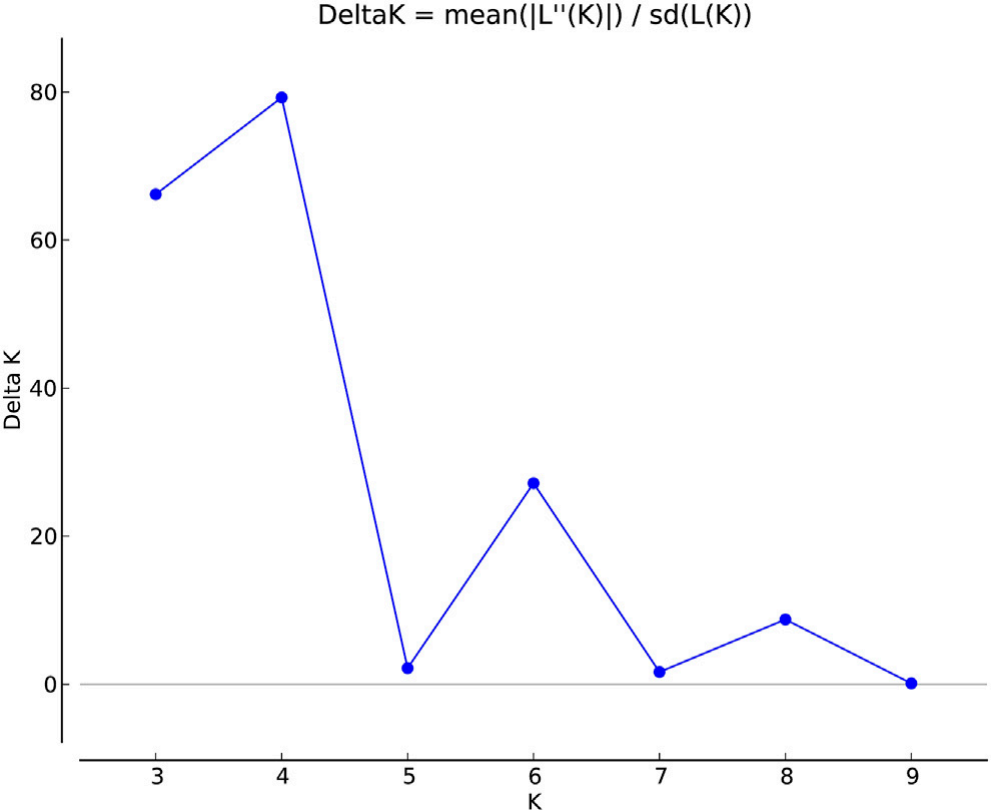
Estimation of population using LnP(D) derived Δk for k from 2 to 10 of a total 2,242 east coast rice collection.

**FIGURE 2:**
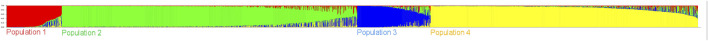
Population structure bar plot of 2,242 east coastal rice accessions.

The genetic link between individual rice accessions was determined using STRUCTURE, a model-based tool. Each accessions’ membership was run from K = 2 to K = 10 (Figure 1). The ultimate number of populations was determined using Structure Harvester (http://taylor0. biology.ucla.edu). The number of populations was found to be four for the entire 2,242 east coast rice collection (Figure 2). Population 1 had 128 pure and 51 admix accessions. Population 2 showed 821 pure and 107 admixed accessions, population 3 showed 157 pure and 78 admixed accessions, and population 4 showed 700 pure and 200 admixed accessions. Most of the aromatic rice accessions got grouped in population 3. Such grouping of aromatic accessions was not seen in the NJ tree. An overview of the state-wise distribution in population shows that population 1 had 60% accessions from Orissa, while in populations 2 and 3, 72% and 93% accessions were from Andhra Pradesh, respectively, fairly exhibiting the dominance of states over population. Population 4 showed around 50% accessions from Tamil Nadu (Figure 2). Likewise, the population mean value of alpha, Fst1, Fst2, Fst3, and Fst4 generated from the model-based approach and allele-freq. divergence among populations (net nucleotide distance), computed using point estimates of population obtained using the model-based approach, is given in Supplementary Table S4 and Supplementary Table S5. Fst values showed good genetic differentiation and acceptable population structure. Venn diagrams between NJ tree and population structure was constructed to find co-linearity between them. There were 21 accessions (0.93%) overlapping between population 1 of STRUCTURE and group 2 of the NJ tree. A total of 254 accessions (11%) were found to be overlapping in population 2 of STRUCTURE and group 2 of the NJ tree. Similarly, 225 accessions (10%) were common in population 3 of STRUCTURE and group 3 of NJ tree, and 754 accessions (33%) were common in population 4 of STRUCTURE and group 3 of the NJ tree. This shows less similarity being observed between groups of the NJ tree and populations of the model-based approach (Supplementary Figures S5–S8).

Clustering of accessions in STRUCTURE was diverse. Apart from obtaining the highest peak at K = 4, additional smaller peaks were obtained at K = 6 and K = 8 (Figure 1), implying that there are subgroups within the four major groups. As a result, an independent STRUCTURE was run with K values from 2 to 10 for all four populations obtained above. Subclustering of population 1 identified the highest peak at K = 4, giving subpopulations named subpopulation 1a, subpopulation 1b, subpopulation 1c, and subpopulation 1d (Supplementary Figures S9, S10). Subclustering of population 2 identified the highest peak at K = 6, giving subpopulations named subpopulation 2a, subpopulation 2b, subpopulation 2c, subpopulation 2d, subpopulation 2e, and subpopulation 2f (Supplementary Figures S11, S12). Subclustering of population 3 also identified the highest peak at K = 6, giving subpopulations named subpopulation 3a, subpopulation 3b, subpopulation 3c, subpopulation 3d, subpopulation 3e, and subpopulation 3f (Supplementary Figures S13, S14). Subclustering of population 4 identified the highest peak at K = 3, giving subpopulations named subpopulation 4a, subpopulation 4b, and subpopulation 4c (Supplementary Figures S15, S16). Grouping in Structure was diverse, and no dominance of states was observed in any of the population. The allocation of accessions from different states and the expected heterozygosity of the 19 subpopulations are shown in Supplementary Table S6. In this table, there are slight variations in the values of the expected heterozygosity ranging between 0.06 and 0.40 with a mean value of 0.22, indicating good genetic diversity in the subpopulation (Luo et al., 2019).

### Population structure of the rice collection of three coastal states (Andhra Pradesh, Orissa, and Tamil Nadu)

Another aspect of population structure was studied to see the clustering of accessions state-wise. Population structure analysis grouped rice collection from Andhra Pradesh into four different populations (Supplementary Figure S17) Population 1 has 150 pure and 40 admix accessions, population 2 has 260 pure and 145 admix accessions, population 3 has 150 pure and 107 admix accessions, and population 4 has 179 pure and 97 admix accessions (Supplementary Figure S18). All aromatic accessions from Andhra Pradesh got grouped in population 3. Rice collection from Orissa got grouped into three populations (Supplementary Figure S19) with population 1 having 62 pure and 24 admix accessions, population 2 having 96 pure and 6 admix accessions, and population 3 having 156 pure and 34 admix accessions (Supplementary figure S20). Rice collection from Tamil Nadu got grouped into five populations (Supplementary Figure S21), population 1 having 44 pure and 20 admix accessions, 73 pure and 41 admix accessions in population 2, 168 pure and 42 admix accessions in population 3, 95 pure and 67 admix accessions in population 4, and 104 pure and 77 admix accessions in population 5 (Supplementary Figure S22). The mean values of alpha, Fst1, Fst2, Fst3, Fst4, Fst5, and Allele-freq. divergence among populations (net nucleotide distance), computed using point estimates of population generated from the model-based approach, are shown in Supplementary Table S7 and Supplementary Table S8, respectively. The values of Fst showed standardized genetic differentiation, suggesting a good population structure. Allele frequency divergence among populations computed using the point estimates of the population also gives about the genetic variation among populations. The values are indicative of the accessions being diverged in the population structure (Luo et al., 2019).

### AMOVA and PCoA of total 2,242 east coast rice collection

The distribution of genetic diversity between and within the populations obtained following STRUCTURE analysis was investigated using AMOVA for total rice accessions (2242). In the first case, four populations were assumed and AMOVA analyses revealed that 29% diversity exists among populations, 20% within individuals, and 51% among individuals of the total east coast rice collection (Supplementary Table S9), while the PCoA plot showed that out of four populations obtained population 3 was getting distinctly separated from the others (Supplementary Table S10; Supplementary Figure S23). In the second case, assuming 19 subpopulations when AMOVA was done there was 30% diversity existing among populations, 21% within individuals, and 49% among individuals of the total east coast rice collection (Supplementary Table S11), while the PCoA plot showed plots with overlapping populations (Supplementary Table S12; Supplementary Figure S24). The AMOVA and PCoA studies confirmed that the actual population number in the case of the east coast collection is only four because assumption of the population of 19 did not show any advantage.

### AMOVA and PCoA of the rice collection coastal states

The AMOVA study of Andhra Pradesh revealed 23% variance within individuals and 30% and 47% variance among populations and among individuals, respectively (Supplementary Table S13; Supplementary figure S25). PCoA plot analysis revealed that population 3 is distinctly isolated from the rest of the populations, and all the aromatic samples from Andhra Pradesh got grouped in to this Supplementary figure S26. Likewise, the AMOVA study of Orissa showed 19%, 68%, and 14% among populations, among individuals and within individuals, respectively (Supplementary Table S13; Supplementary figure S27). The PCoA plot showed mixing of three populations (Supplementary figure S28). The AMOVA study of Tamil Nadu showed 24%, 53%, and 23% variance among populations, among individuals, and within individuals, respectively (Supplementary Table S13; Supplementary figure S29). The PCoA plot revealed slightly isolated population 3 (Supplementary figure S30); however, there was intermixing between populations 1, 2, 4, and 5. Principal coordinate analyses of rice collection of coastal states, with percentage of variation explained by the first three axes, are summarized in Supplementary Table S14.

### Generation of the core set

**FIGURE 3:**
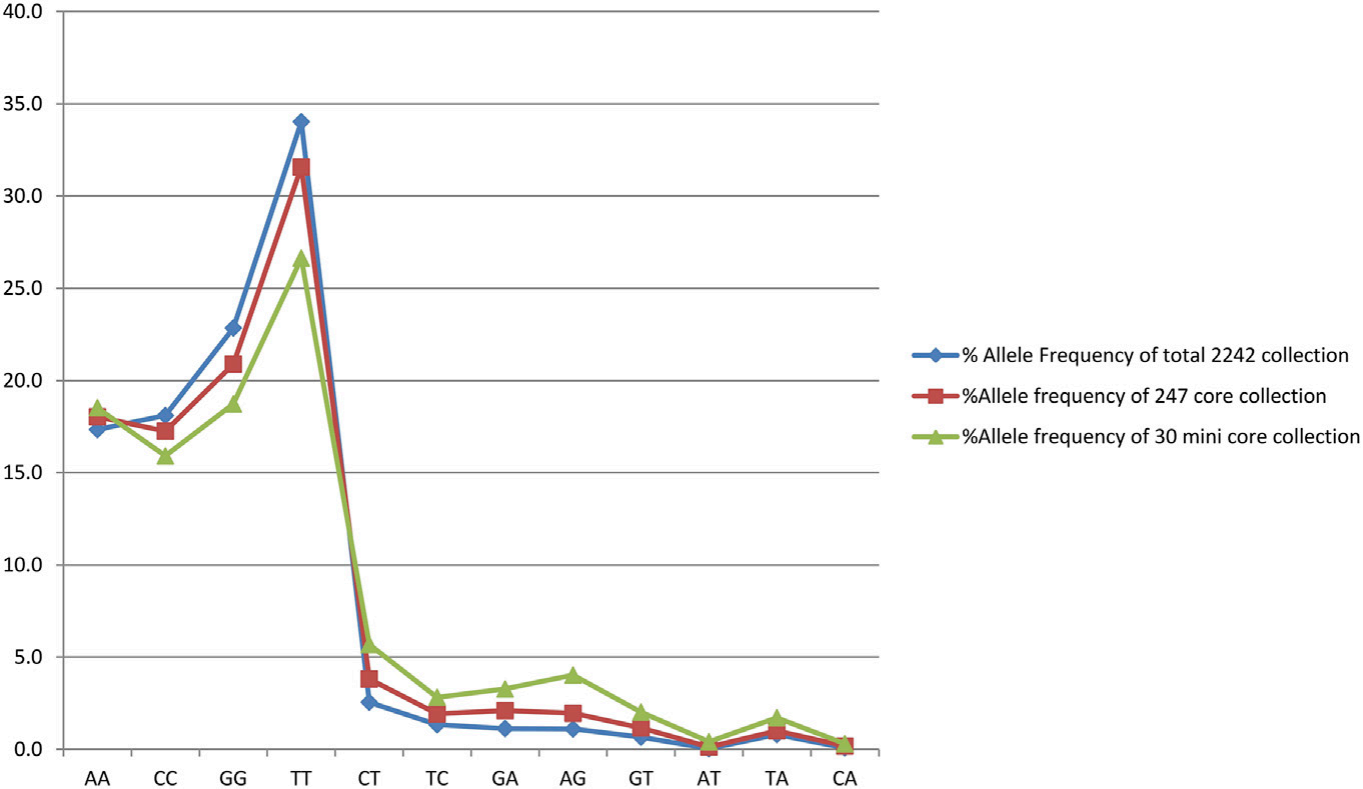
Graph showing the allele frequency of total east coast collection, core collection, and mini core collection.

**FIGURE 4:**
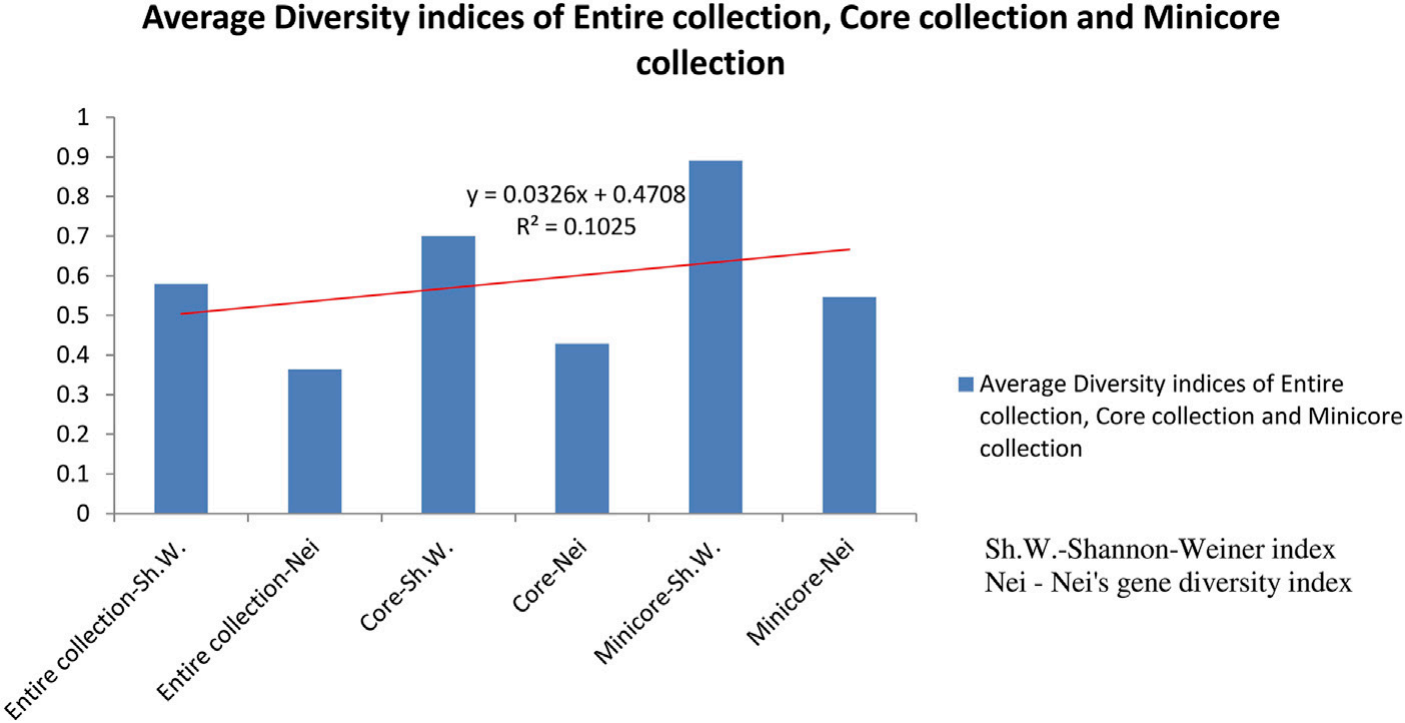
Graph showing average diversity indices of entire collection, core collection, and mini core collection.

Out of 2,242 rice accessions studied, a core set of 247 accessions (i.e., 126 accessions from Andhra Pradesh, 45 accessions from Orissa, and 76 accessions from Tamil Nadu) and a mini core set of 30 accessions were selected using POWERCORE (Supplementary Tables S2, S3). Thirty-six SNP markers produced nine allele types, four of which were homozygous and six were heterozygous (three transitions and two transversions). We found no C/G- or G/C-type substitutions in our research. Allele frequency was determined for all three state collections and their core sets. The study of allele frequency revealed that no alleles were lost in the resulting core set and they were 99.9% similar. The same has been plotted in line plots for the total 2,242 in the east coast collection as well as for the core sets (Figure 3). These results were also in concordance with the results obtained by PowerCore where the Shannon’s diversity index and Nei’s gene diversity showed an increasing trend starting from the entire 2,242 collection to 247 core to 30 mini core collections (Figure 4). The 247 core accessions represent 11.01% of the entire collection, and the 30 mini core set represents 12.14% of the core collection (Supplementary Figure S31). Thus, we have been able to fulfill the recommended size required for a perfect core collection, which is between 5% and 20% of the original size (Bisht et al., 1998).

### Genetic diversity of the core set and the mini core set

**FIGURE 5:**
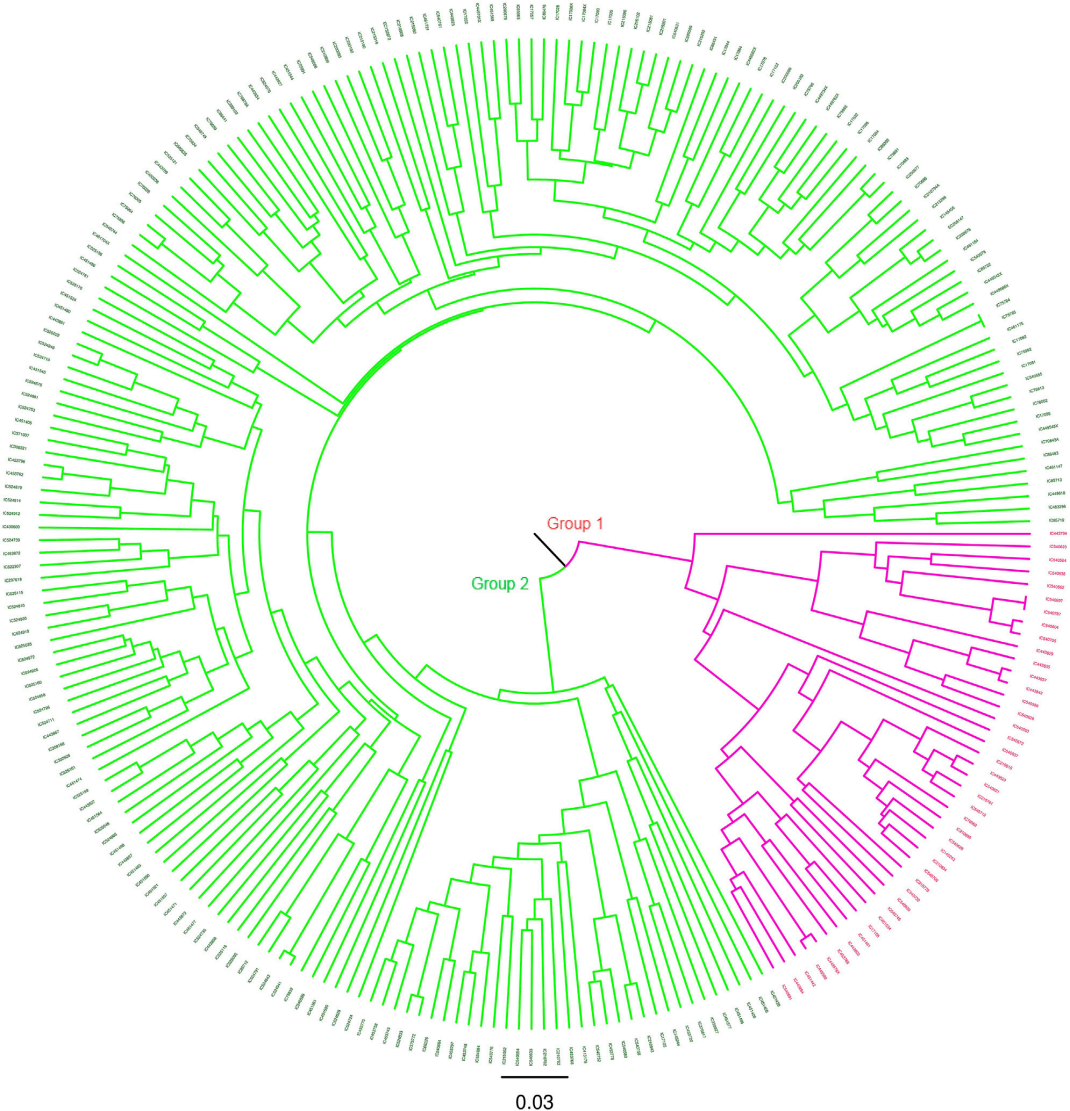
NJ tree of the 247 east coast core collection.

**FIGURE 6:**
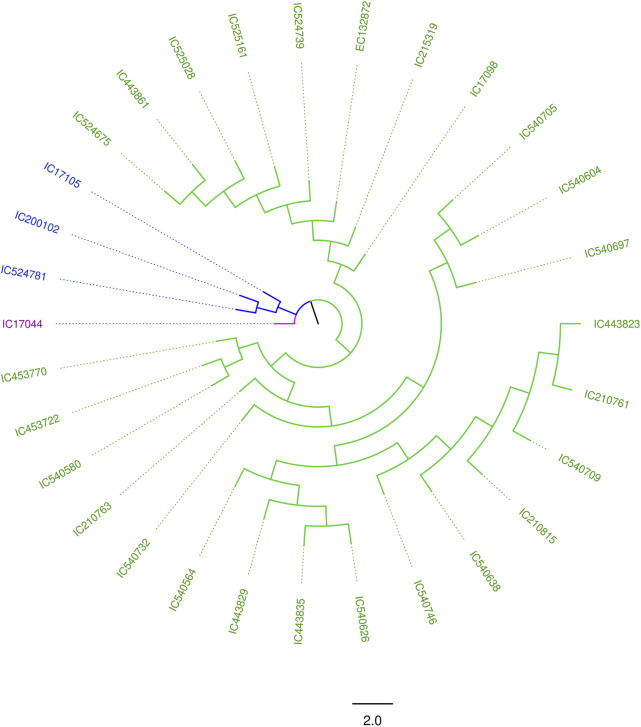
NJ tree of the east coast mini core set.

The degree of genetic diversity of the core set was studied to find the degree of genetic diversity captured from the overall coastal collection. Major allele frequency, gene diversity, heterozygosity, and PIC were observed to be 0.75, 0.34, 0.12, and 0.27 respectively (Table 2). The values of genetic diversity of the mini core set were approximately similar to the core, as shown in Table 2. A greater value genetic diversity has been observed in case of core/mini core than that of the total east coast rice collection. Hence, definitely the east coast core collection developed has rich and diverse representatives having good diversity parameters. The NJ tree showed two distinct groups in the core set and three groups in the mini core set (Figures 5, 6).

### Population structure of core set

**FIGURE 7:**
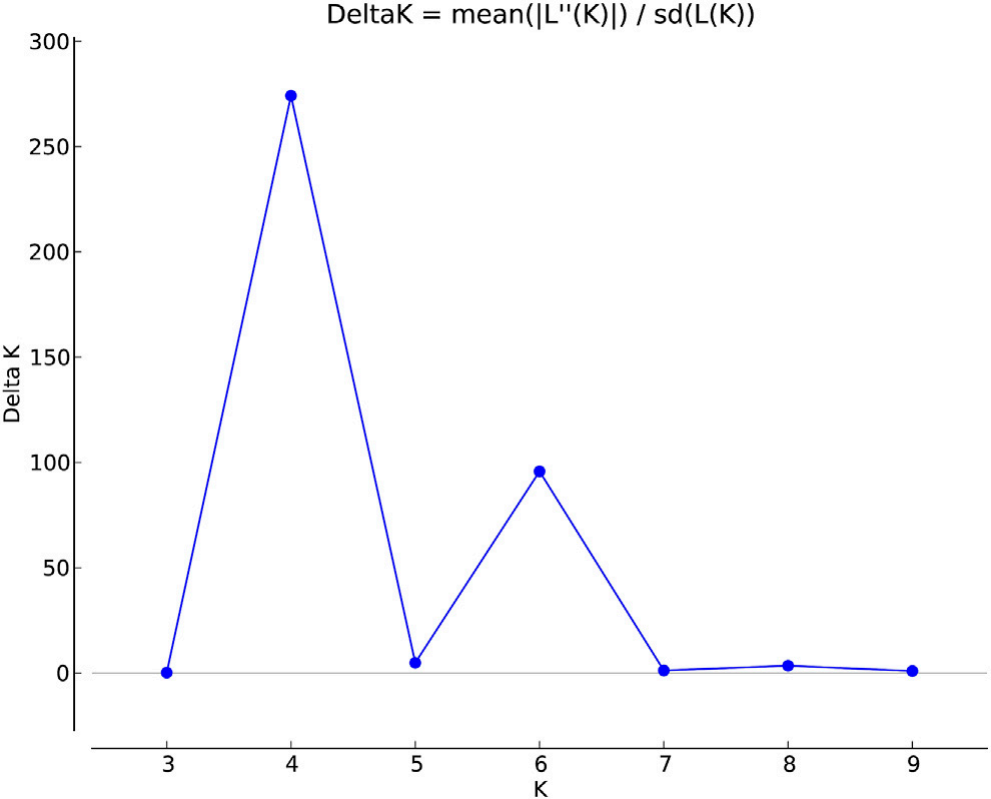
Estimation of population using LnP(D) derived Δk for k from 2 to 10 of the east coast core collection.

**FIGURE 8:**
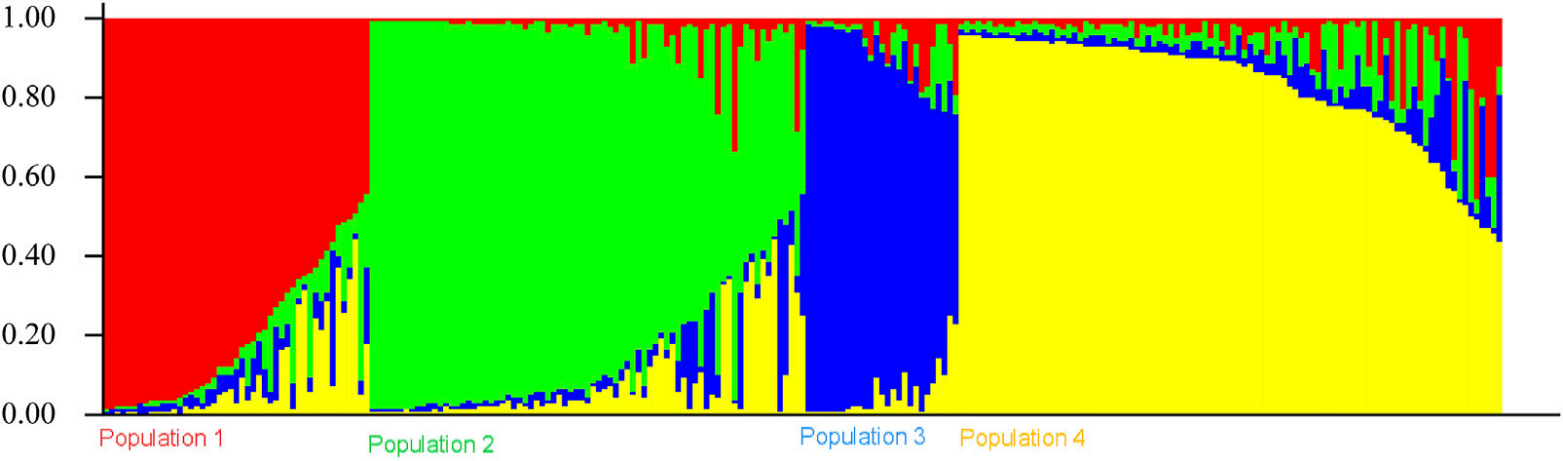
Population structure bar plot of the east coast core collection.

The population structure grouped the core set accessions into four populations (Figures 7, 8). The STRUCTURE bar plot showed population 1 having 27 pure and 20 admixed accessions, population 2 having 55 pure and 22 admixed accessions, population 3 having 20 pure and 7 admixed accessions, and population 4 having 60 pure and 36 admixed accessions (Figure 8). The mean value of alpha (0.10) for the core collection is greater than the mean value of alpha for the total collection (0.06). An alpha value close to zero means that individuals are essentially from different populations (Evanno et al., 2005). In our case, a 0.10 value of alpha in the core collection as compared to the 0.06 value of alpha in the total collection signifies more admixed individuals in the total collection. Allele-freq. divergence among populations (net nucleotide distance), computed using point estimates of population (core collection), are given in Supplementary Table S15 and Supplementary Table S16.

### AMOVA and PCoA of the core set

The AMOVA study of the core collection revealed 29% variance within individuals as well as among population and 42% variance among individuals (Supplementary Table S17; Supplementary Figure S32). The PCoA plot showed populations being scattered in different quadrants (Supplementary Figure S33; Supplementary Table S18).

### Kinship analysis of the core set collection

**FIGURE 9:**
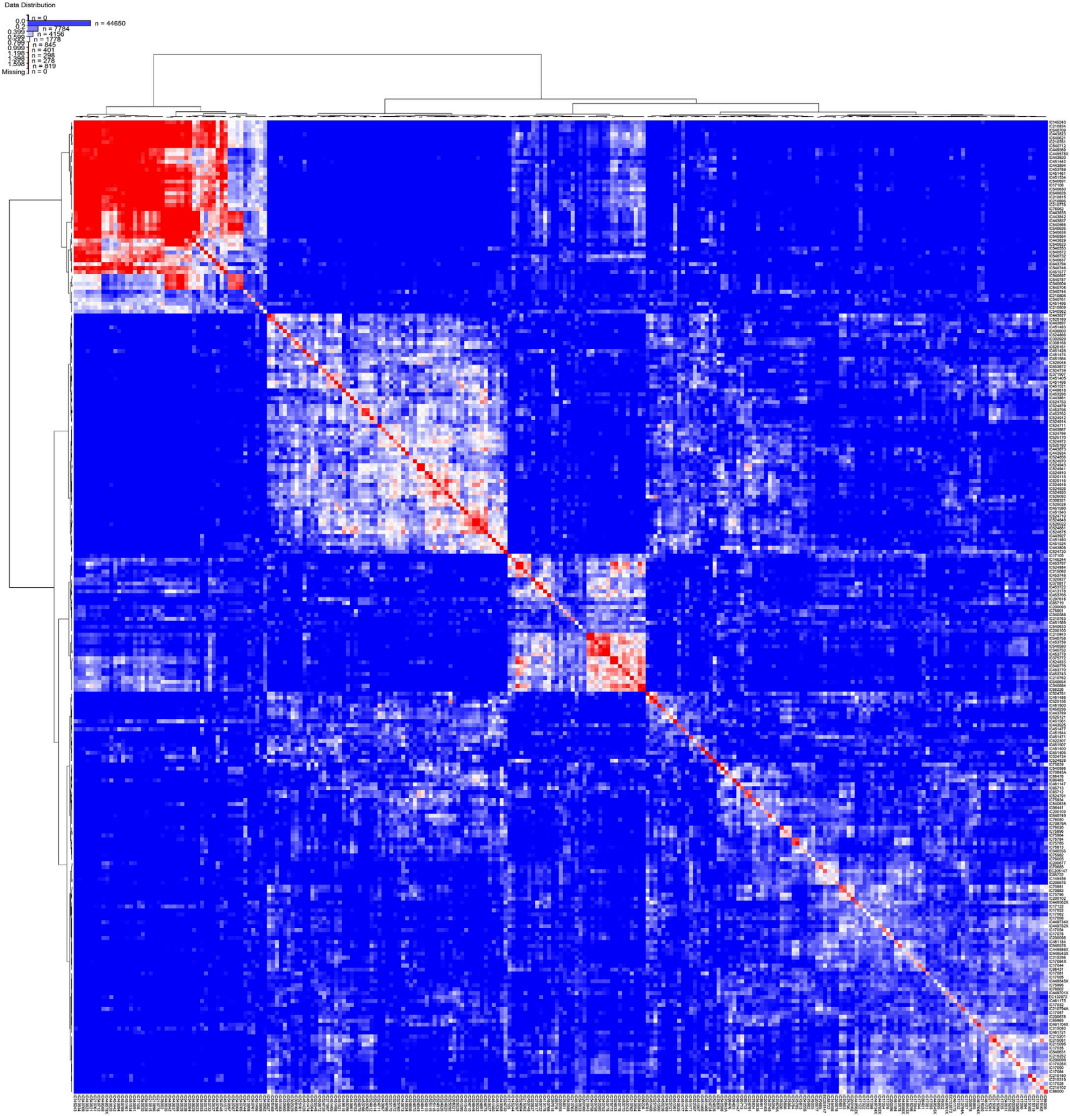
Structured heat map showing the relatedness of rice accessions from core collection.

Kinship analysis of the core set showed that more than 50% of the samples had a kinship value less than zero, and less than 10% of the accessions had kinship values between 0.5 and 0.75 (Supplementary Figure S34). The kinship index and clustered heat map showed more diversity in the core collection because clustering based on this map was more heterogeneous, which indicates that maximum unique genotypes have been selected in the core collection (Figure 9).

### Validation of SNP markers in coastal rice collection and northeast rice collection

**FIGURE 10:**
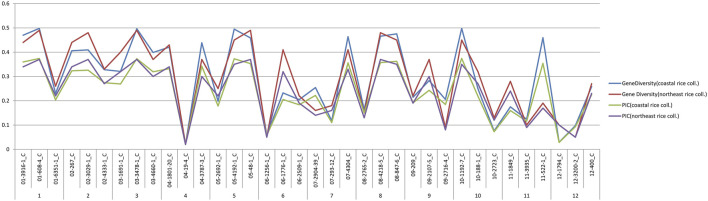
Comparison of gene diversity and PIC of east coast rice and northeast rice collection.

A comparative analysis of genetic diversity parameters, i.e., PIC and gene diversity between the coastal rice and northeast rice collection (Roy Choudhury et al., 2014), was done and are shown in Figure 10. On comparing the values, in both collections it was observed that in the case of the PIC, the same markers had given the maximum and minimum values in both collections (i.e., coastal rice collection and northeast rice collection**)**. This means that markers 01-608-4_C and 03-3478-1_C had given the maximum PIC value of 0.37 in both the collections and marker 04-19-4_C had given the minimum PIC value of 0.01 in both collections. Similarly, markers 01-608-4_C and 03-3478-1_C had displayed the highest gene diversity with a value of 0.49 across both collections and a minimum value of gene diversity 0.02 with marker 04-19-4_C across both collections. Generally, line graphs for PIC and gene diversity were overlapping for both collections except at certain points where deviations were observed. For example, marker 03-1691-1_C gave a gene diversity value of 0.32 in the coastal rice collection and 0.4 in the northeast rice collection. The same marker gave a PIC value of 0.26 in the coastal rice collection and 0.32 in the northeast rice collection. Marker 11-522-1_C gave a gene diversity value of 0.45 in the coastal collection and 0.19 across the northeast collection; also, this marker 11-522-1_C gave PIC values of 0.35 and 0.17 across the coastal rice and northeast rice collections. A subsequent analysis between major allele frequency and heterozygosity from the current study and with the northeast rice collection was also evaluated (Supplementary figure S35). The values for major allele frequency and heterozygosity were overlapping except at few points where deviation was observed. For example, marker 04-1801-20_C gave heterozygosity values of 0.42 and 0.28 in coastal collection and northeast collection, respectively depicting a small amount of deviation. Similarly, marker 11-522-1_C gave major allele frequency values of 0.64 and 0.89 in the coastal collection and northeast collection, respectively, again depicting slight deviations. The validation of the same set of SNP markers (36-plex assay) in different collections (northeast and east coast collection) shows that they are very effective in deciphering the genetic diversity parameters in both the collections.

## Discussion

The east coast collection of rice germplasm available at the National Genebank, NBPGR, New Delhi, is a valuable collection of rice accessions for the assessment of genetic diversity and other important traits. Rice accessions from different collections have served as and continued to act as sources of genes for desired qualities, contributing to the variety developments that have been reported (Choudhury et al., 2013; Das et al., 2013). Regular floods hit coastal areas and have saline soils, and various other constraints make them a fragile ecosystem with lower productivity and slow trend in growth rate (Amanullah et al., 2007). Therefore, a coastal core collection would be an appropriate measure to conserve rice in these areas for better management studies. Despite advances in genomics, the Indian rice collection has remained uncharacterized at the molecular level in terms of genetic diversity and population structure. This has been a key stumbling block in their ability to use and develop superior cultivars. In this study, effort has been made to characterize the east coast rice collection available at the National Genebank, NBPGR, New Delhi, using 36 SNP markers to enhance genome wide studies in rice. These unlinked SNP markers, which were generated and used in diverse studies, are located on the short arm, centromeric region, and long arm of all 12 rice chromosomes. As discussed, a state-wise study of the east coast collection of rice showed interesting results. A total of 72 alleles were amplified with 2 alleles per locus. The average PIC values ranged from 0.20 for Tamil Nadu, 0.21 for Orissa, and 0.25 for Andhra Pradesh. The values observed are concurrent with those observed by Singh et al. (2013) on 375 rice varieties (0.25) and Roy Choudhury et al. (2014) on the northeast rice collection (0.23) using SNP markers. Chen et al. (2017) observed PIC values of 0.27 on *Ziziphus jujuba* Mill, China’s most important fruit species, and 0.29 reported by Luo et al. (2019) on *Camelina sativa* using SNP markers. A PIC value of 0.4 was reported by Mourad et al. (2020) while they were studying the genetic diversity, population structure, and linkage disequilibrium in the spring wheat core collection, which is higher than that reported in the present study. The PIC value is generally high when SSR markers are used as observed by Rashmi et al. (2017) in 65 rice accessions (0.38) characterized using SSR markers. Pathaichindachote et al. (2019) reported a PIC value of 0.56 in 167 Thai and exotic rice varieties using 49 SSR markers and a PIC value of 0.63 reported by Jasim Aljumaili et al. (2018) on 50 aromatic rice accessions with 32 SSR markers. A mean PIC value of 0.61 has been reported by Suvi et al. (2020) while they were accessing the genetic diversity and population structure of 54 rice accessions using 14 SSR markers. Tarang et al. (2020) reported a PIC value of 0.92 with 60 microsatellite markers of 63 rice genotypes in Central and West Asia. PIC values and expected heterozygosity (He, also called gene diversity) are both indices of genetic diversity among genotypes in breeding populations. This also reveals the evolutionary pressure on the alleles as well as the mutation rate a locus may have experienced over time (Botstein et al., 1980; Shete et al., 2000; Luo et al., 2019). In our study, the average gene diversity was observed to be 0.24, 0.26, and 0.30 for Tamil Nadu, Orissa, and Andhra Pradesh, respectively (Table 2). As a result, the overall gene diversity value was slightly higher than the PIC value, which was expected. The PIC values will always be lower than gene diversity and will become closer to gene diversity as more alleles are added and with rising evenness of allele frequencies (Shete et al., 2000). PIC values are limited to 0.5 due to the biallelic nature of the SNPs (where the two alleles have identical frequencies) (Eltaher et al., 2018) and could possibly be attributable to low mutation rates in SNPs (Coates et al., 2009; Eltaher et al., 2018; Luo et al., 2019). There are some accessions in the total list of accessions which have the same IC numbers followed by X and P; these are accessions which were collected at two different periods from the same areas, hence denoted by X or P. It has been distinctly noticed that these accessions and their original counterparts did not give the same results with SNP markers; this could be due to the high evolutionary drive during collection at different periods. (Kasso and BalaKrishnan, 2013).

The genetic distances were estimated, and the dissimilarity matrix was used to build the NJ tree. The NJ tree of the 2,242 coastal samples showed three major groups. However, nothing very captivating was observed in the clusters formed. Such widely overlapped groups in the NJ tree has also been reported by Xu et al. (2016) on *indica* rice.

Initially, the population structure of the overall east coast collections revealed four populations. A subsequent population structure analysis gave 19 populations altogether. A weak population structure and low relatedness as revealed in kinship analysis between the east coast rice accessions and the core accessions support the statement by Nachimuthu et al. (2015) that these are critical factors to circumventing spurious data hindering the downstream study (). In the present study, the NJ tree, Bayesian-based STRUCTURE, and AMOVA and PCoA did not show any consensus clustering that could be highlighted. Similar results were also observed by Ambreen et al. in 2018. Also, a population structure study on the rice collection of the east coast states revealed four, three, and five populations for Andhra Pradesh, Orissa, and Tamil Nadu, respectively. In case of Andhra Pradesh, population structure showed a conspicuous grouping of aromatic samples in population 3. Similar type of grouping was reported in basmati rice by Civáň et al. (2019). Admixtures were observed, which suggests that besides pure lines there are samples which are heterogeneous in nature. The mean values of alpha ranged from 0.04 for Orissa, 0.05 for Tamil Nadu, and 0.10 for Arunachal Pradesh (Supplementary Table S4). When the alpha value approaches zero, it means that the majority of individuals are from distinct populations (Li et al., 2014). The values of Fst correspond to a standardized genetic differentiation, suggesting an acceptable population structure. The STRUCTURE analysis indicated good genetic diversity among the rice accessions of the east coast collection. The presence four populations were confirmed by model-based analysis. This method has been used extensively by Edae et al. (2014) to explore association mapping.

The advanced M strategy with minimum redundancy and heuristic approach was used for east coast core collection. The minimum redundancy is required for increasing allelic richness in the core collection; hence, accessions need to be of unique allelic combinations and an unstructured population (Ambreen et al., 2018). A kinship analysis study of the east coast core collection demonstrated a low amount of genetic relatedness, meeting the key condition of an ideal core collection as well as an idealistic association panel (Kumar et al., 2020). Thus, this coastal core set qualifies all the benchmarks of a standard core set.

While validating our results with the northeast rice collection (Roy Choudhury et al., 2014), markers 01-608-4_C and 03-3478-1_C were found to give the highest PIC value of 0.37, while marker 04-19-4_C was least informative giving the lowest PIC of 0.01 in both collections. Marker 01-608-4_C is a locus of evolutionary conserved genes from the saponin family of proteins, which is involved in the sphingolipid metabolic process and active in extracellular space (Bruhn, 2005); in the present study, this marker is highly conserved yet highly polymorphic. However, marker 03-3478-1_C, which is a locus of the GRP (gibberellin-regulated protein) family and an evolutionary conserved gene involved in plants’ defense mechanism as well as in growth (Inomata, 2020), has not been observed polymorphic in both collections, which is contradictory to earlier an report by Mukesh Jain et al. (2014) in rice.

Validation of the same set of SNP markers on two collections (northeast rice and east coast rice collection) has established that the 36-plex SNP assay is sufficient and efficient for initial diversity analysis and core development. Hence, this 36-plex SNP assay can be exploited by researchers for the genetic diversity study and development of core based on their own collections, thus accelerating their breeding program.

## Conclusion

This is the first study where India’s east coast rice collections were characterized using SNP markers. The genetic diversity and population structure were studied, and core and mini core collections with maximum diversity and minimum redundancy were developed. A total of 2,242 east coast rice accessions from three different states of India, i.e., Andhra Pradesh, Orissa, and Tamil Nadu, have been characterized, and a wide range of gene diversity and PIC was observed. A phylogenetic analysis of the total east coast rice collection revealed three groups, and a population structure analysis revealed four populations. The 36-SNP assay used in this study was validated by comparing the genetic diversity parameters (gene diversity, PIC, major allele frequency, and heterozygosity) across two different rice collections, i.e., east coastal rice and northeast rice collection, and it was observed the these markers were sufficient to decipher all genetic parameters very efficiently; hence, they can be effectively utilized for core development and diversity study of different rice genotypes.

## Data Availability

The original contributions presented in the study are included in the article/Supplementary Material; further inquiries can be directed to the corresponding author.

## Supplementary Material

Supplementary Figure S1 Neighbor joining tree of the total 2242 east coast rice collection

Supplementary Figure S2 Neighbor joining tree of 1133 rice collection of Andhra Pradesh

Supplementary Figure S3 Neighbor joining tree of 378 rice collection of Orissa

Supplementary Figure S4 Neighbor joining tree of 731 rice collection of Tamil Nadu

Supplementary Figure S5 Venn diagram showing co-linearity between all three groups of neighbor joining tree and population 1 of population structure of total east coast rice collection

Supplementary Figure S6 Venn diagram showing co-linearity between all three groups of neighbor joining tree and population 2 of population structure of total east coast rice collection

Supplementary Figure S7 Venn diagram showing co-linearity between all three groups of neighbor joining tree and population 3 of population structure of total east coast rice collection

Supplementary Figure S8 Venn diagram showing co- linearity between all three groups of neighbor joining tree and population 4 of population structure of total east coast rice collection

Supplementary Figure S9 Hierarchical population structure analysis of subpopulation of population 1 total east coast rice collection

Supplementary Figure S10 Bar plot of population 1 highlighting the sub populations in east coast rice collection

Supplementary Figure S11 Hierarchical population structure analysis of subpopulation of population 2 total east coast rice collection

Supplementary Figure 12 Bar plot of population 2 highlighting the sub populations in east coast rice collection

Supplementary Figure S13 Hierarchical population structure analysis of subpopulation of population 3 total east coast rice collection

Supplementary Figure S14 Bar plot of population 3 highlighting the sub populations in east coast rice collection

Supplementary Figure S15 Hierarchical population structure analysis of subpopulation of population 4 total east coast rice collection

Supplementary Figure S16 Bar plot of population 4 highlighting the sub populations in east coast rice collection

Supplementary Figure S17 Estimation of population using LnP(D) derived Δk for k from 2 to10 of Andhra Pradesh rice collection

Supplementary Figure S18 Bar plot of population structure of Andhra Pradesh rice collection

Supplementary Figure S19 Estimation of population using LnP(D) derived Δk for k from 2 to10 of Orissa rice collection

Supplementary Figure S20 Bar plot of population structure of Orissa rice collection

Supplementary Figure S21 Estimation of population using LnP(D) derived Δk for k from 2 to10 of Tamil Nadu rice collection

Supplementary Figure S22 Bar plot of population structure of Tamil Nadu rice collection

Supplementary Figure S23 Scattered plot (PCoA) of total east coast rice accessions (2242) (among 4 populations)

Supplementary Figure S24 Scattered plot (PCoA) of total east coast rice accessions (2242) (among 19 sub populations)

Supplementary Figure S25 Pie chart showing percentage of molecular variance of rice collection of Andhra Pradesh

Supplementary Figure S26 Scattered plot (PCoA) of rice collection of Andhra Pradesh

Supplementary Figure S27 Pie chart showing percentage of molecular variance of rice collection of Orissa

Supplementary Figure S28 Scattered plot (PCoA) of rice collection of Orissa

Supplementary figure S29 Pie chart showing percentage of molecular variance of rice collection of Tamil Nadu

Supplementary Figure S30 Scattered plot (PCoA) of rice collection of Tamil Nadu

Supplementary Figure S31 Venn Diagram showing the distribution of accessions in the east coast core and the mini-core collection

Supplementary Figure S32 Pie chart showing percentage of molecular variance of the east coast rice core set (247)

Supplementary Figure S33 Scattered plot (PCoA) of the east coast rice core set (247)

Supplementary Figure S34 Histogram showing the kinship status of rice accessions in the east coast core collection

Supplementary Figure S35 Comparative analysis of heterozygosity and major allele frequency values across eat coast rice and north-east rice collection
