# Supplementary material for: Identification of a Diverse Core Set Panel of Rice From the East Coast Region of India Using SNP Markers
Source: Front Genet. 2021 Nov 25;12:726152. doi: 10.3389/fgene.2021.726152 (PMC8655924; doi:10.3389/fgene.2021.726152)
Supplement: Supplementary file 1 [file DataSheet2.pdf]

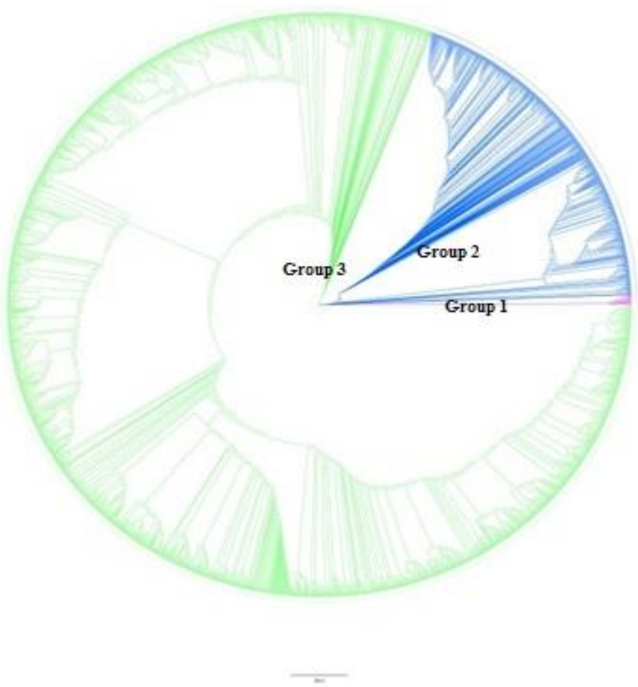

Supplementary Figure S1. Neighbors joining tree of the total 2242 east coast rice collection

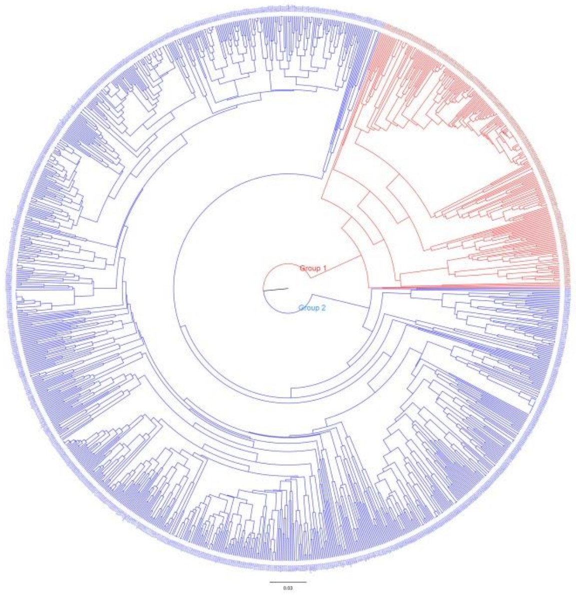

Supplementary Figure S2. Neighbors joining tree of 1133 rice collection of Andhra Pradesh

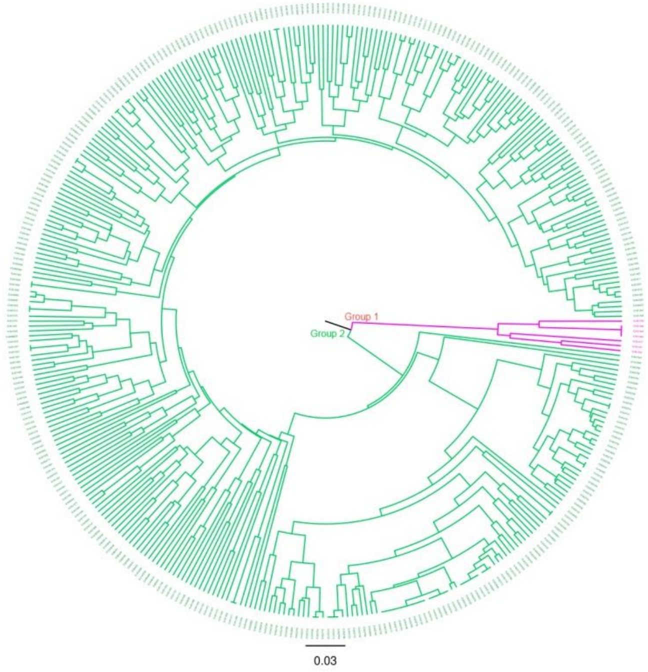

Supplementary Figure S3. Neighbors joining tree of 378 rice collection of Orissa

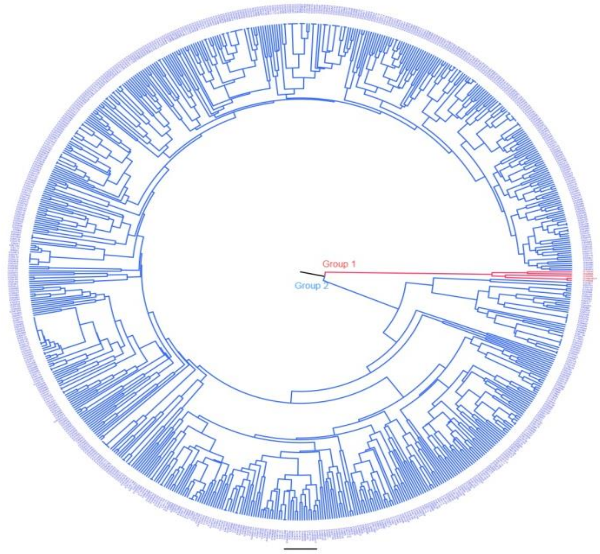

Supplementary Figure S4. Neighbors joining tree of 731 rice collection of Tamil Nadu

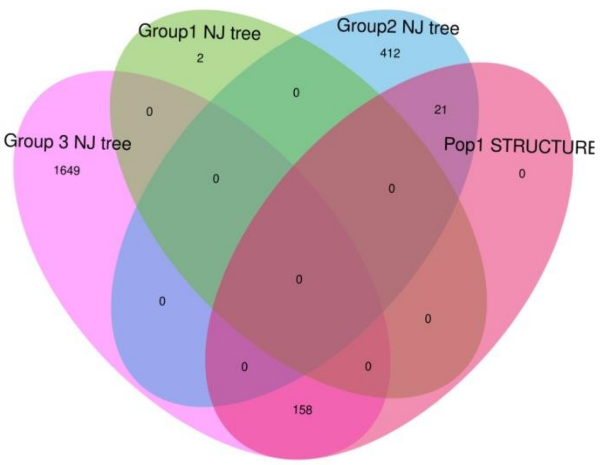

Supplementary Figure S5. Venn diagram showing co-linearity between all three groups of neighbors joining tree and population 1 of population structure of total east coast rice collection

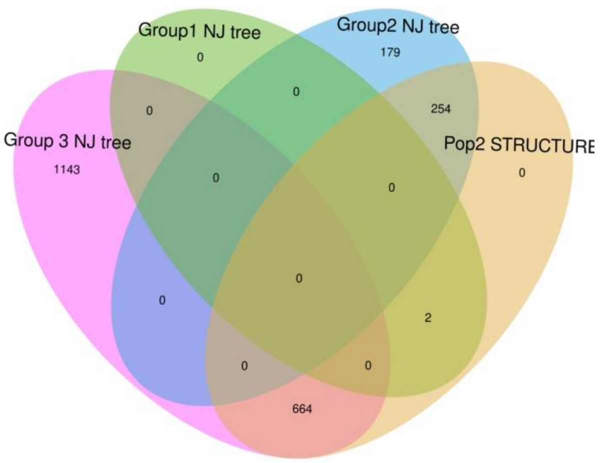

Supplementary Figure S6. Venn diagram showing co-linearity between all three groups of neighbors joining tree and population 2 of population structure of total east coast rice collection

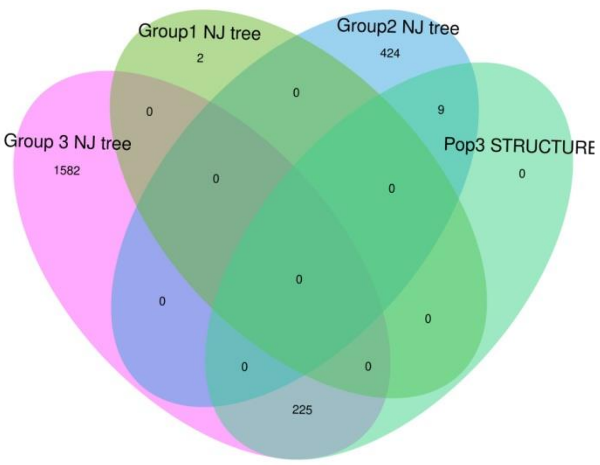

Supplementary Figure S7. Venn diagram showing co-linearity between all three groups of neighbors joining tree and population 3 of population structure of total east coast rice collection

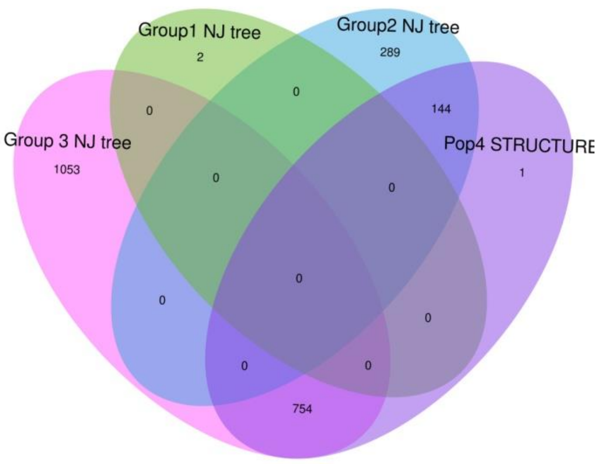

Supplementary Figure S8. Venn diagram showing co-linearity between all three groups of neighbors joining tree and population 4 of population structure of total east coast rice collection

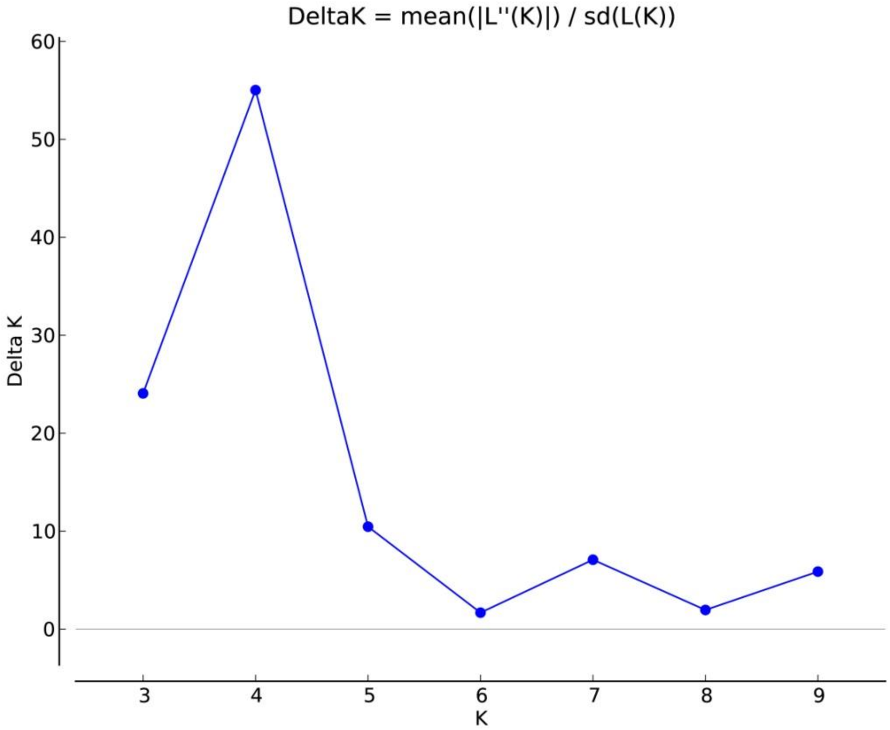

Supplementary Figure S9 Population structure analysis of subpopulation of population 1 total east coast rice collection

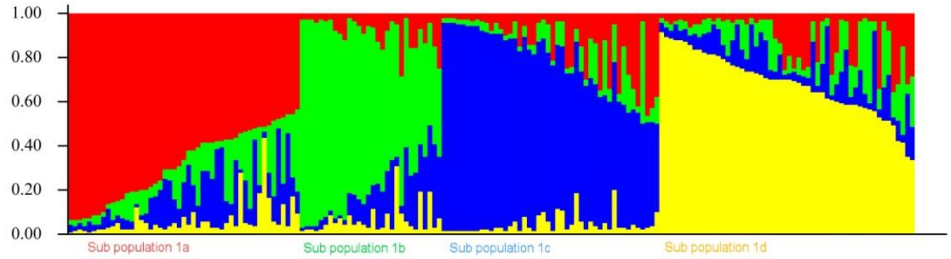

Supplementary Figure S10 Bar plot of population 1 highlighting the sub populations in east coast rice collection

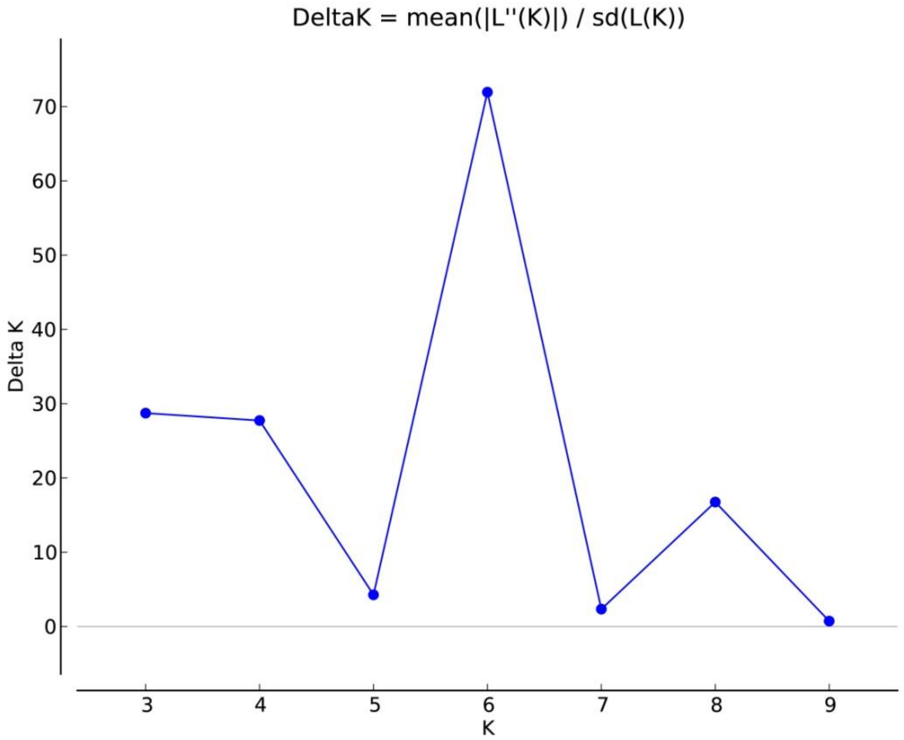

Supplementary Figure S11 Population structure analysis of subpopulation of population 2 total east coast rice collection

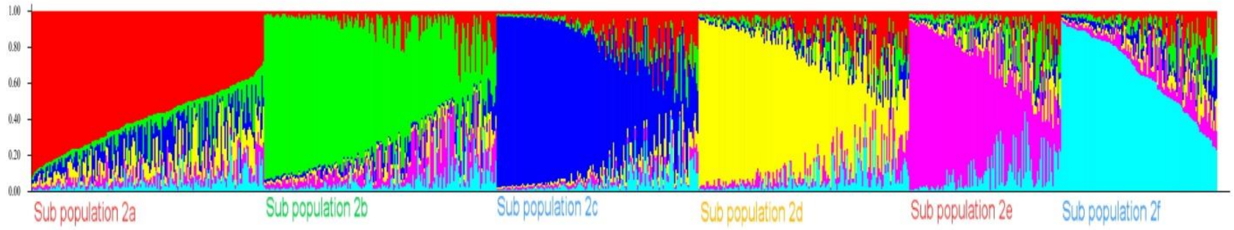

Supplementary Figure S12 Bar plot of population 2 highlighting the sub populations in east coast rice collection

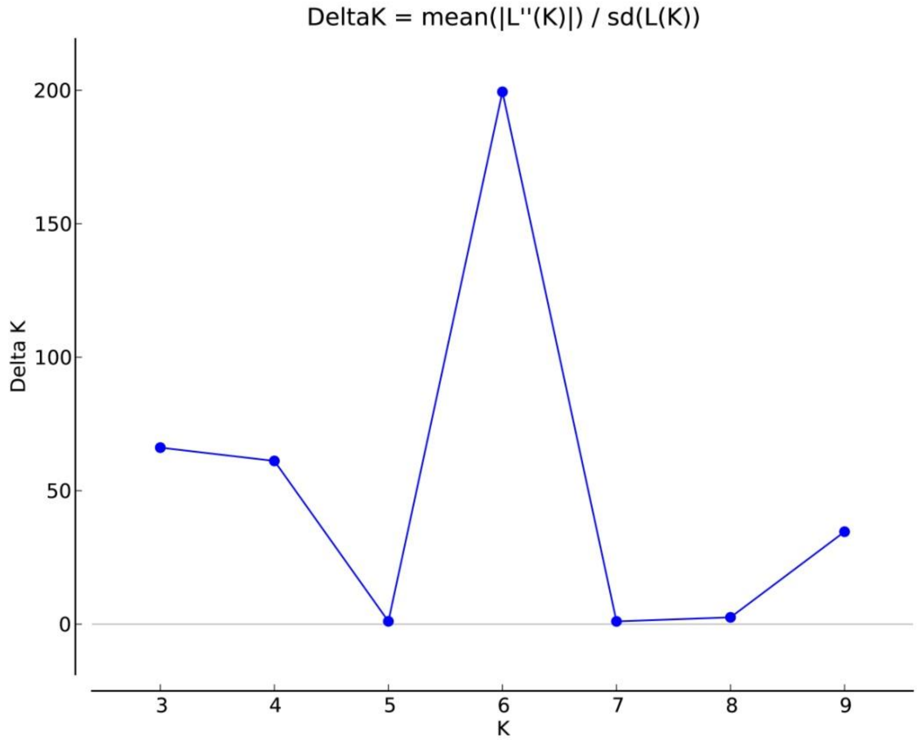

Supplementary Figure S13 Population structure analysis of subpopulation of population 3 total east coast rice collection

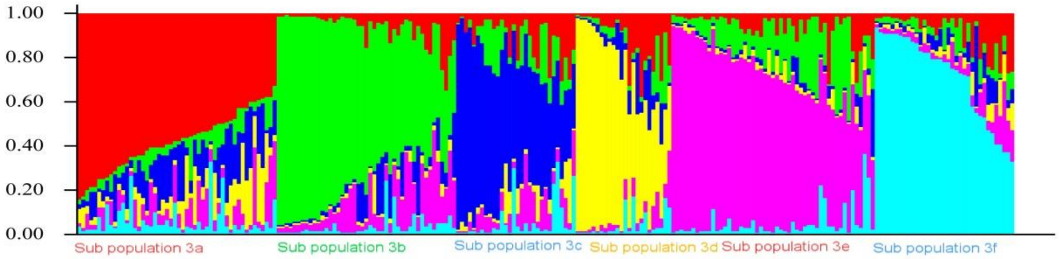

Supplementary Figure S14 Bar plot of population 3 highlighting the sub populations in east coast rice collection

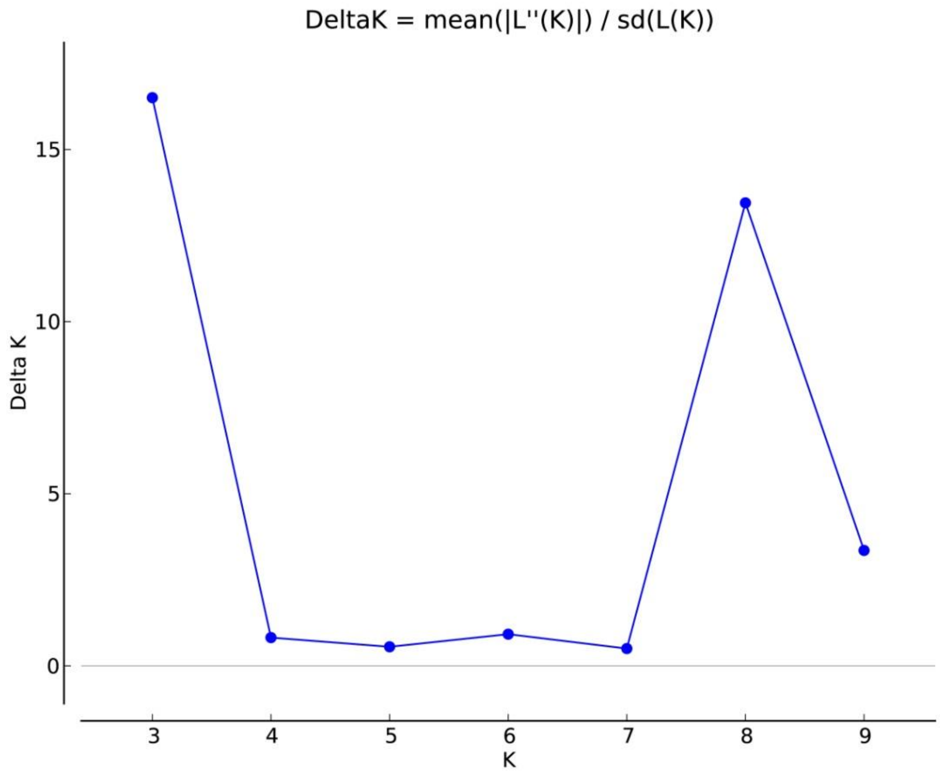

Supplementary Figure S15 Population structure analysis of subpopulation of population 4 total east coast rice collection

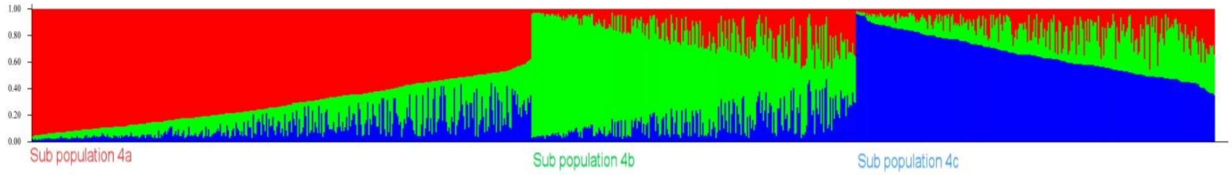

Supplementary Figure S16 Bar plot of population 4 highlighting the sub populations in east coast rice collection

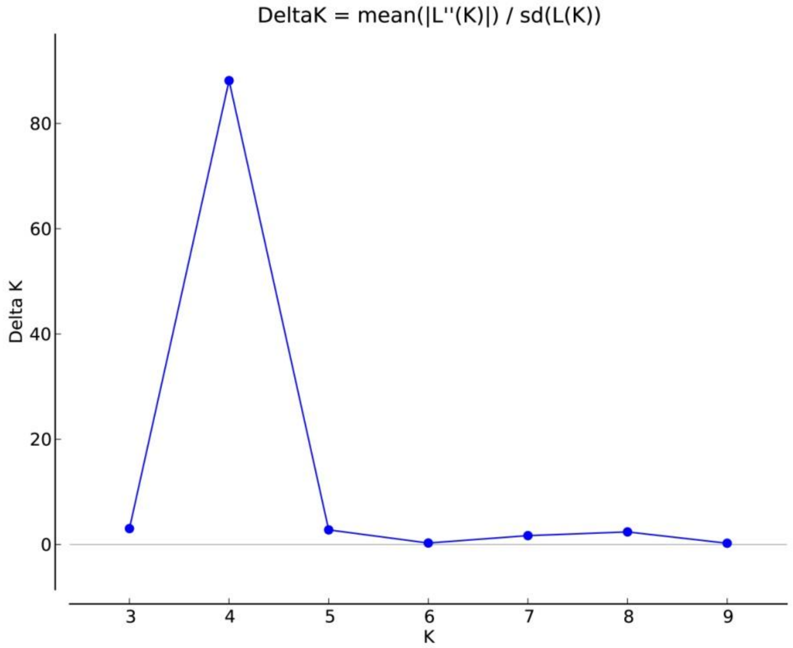

Supplementary Figure S17 Estimation of population using LnP(D) derived Δk for k from 2 to10 of Andhra Pradesh rice collection

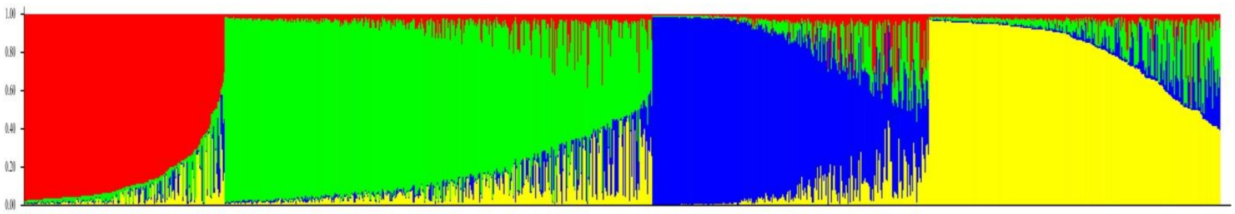

Supplementary figure S18 Bar plot of population structure of Andhra Pradesh rice collection

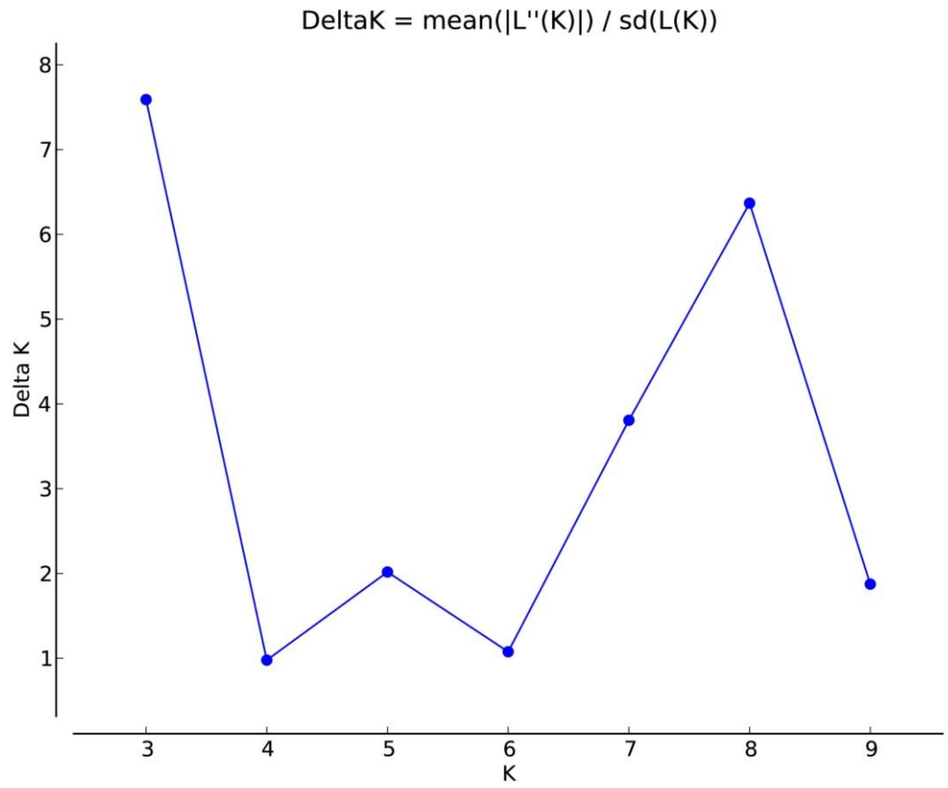

Supplementary figure S19 Estimation of population using LnP(D) derived Δk for k from 2 to10 of Orissa rice collection

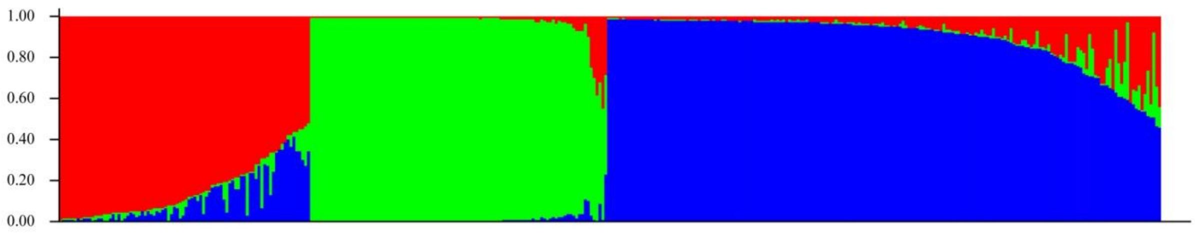

Supplementary figure S20 Bar plot of population structure of Orissa rice collection

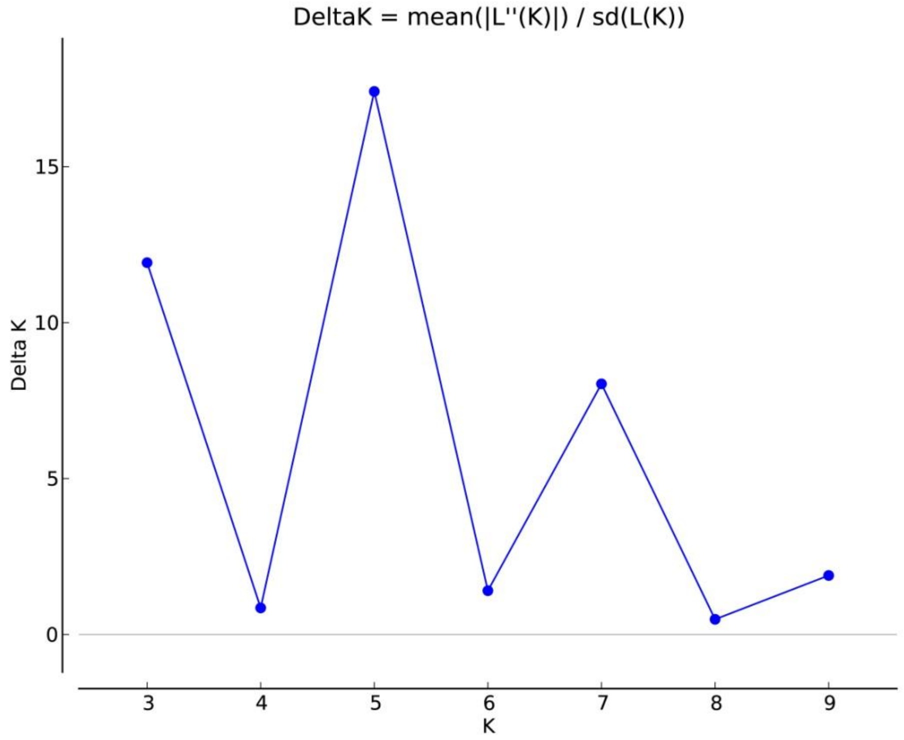

Supplementary figure S21 Estimation of population using LnP(D) derived Δk for k from 2 to10 of Tamil Nadu rice collection

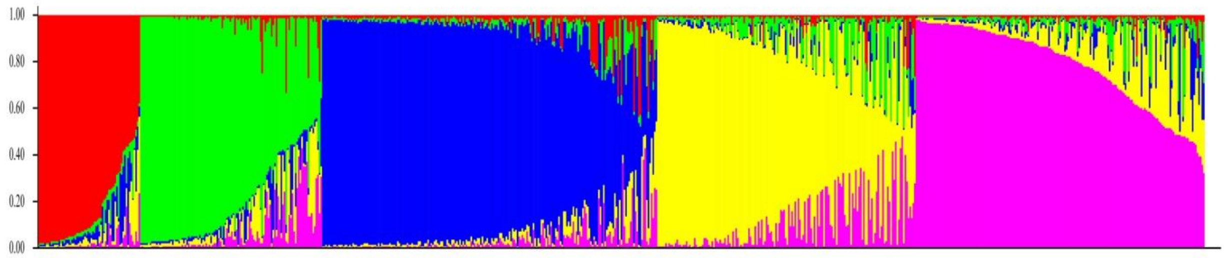

Supplementary figure S22 Bar plot of population structure of Tamil Nadu rice collection

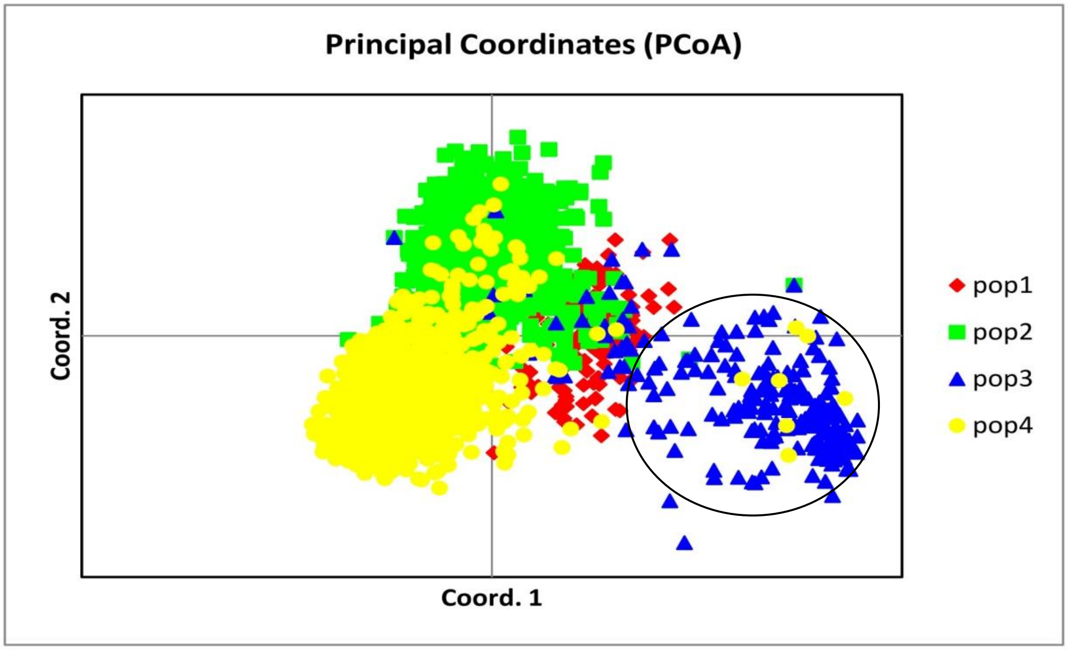

Supplementary figure S23 PCoA analysis total east coast rice accessions (2242) (assuming 4 populations)

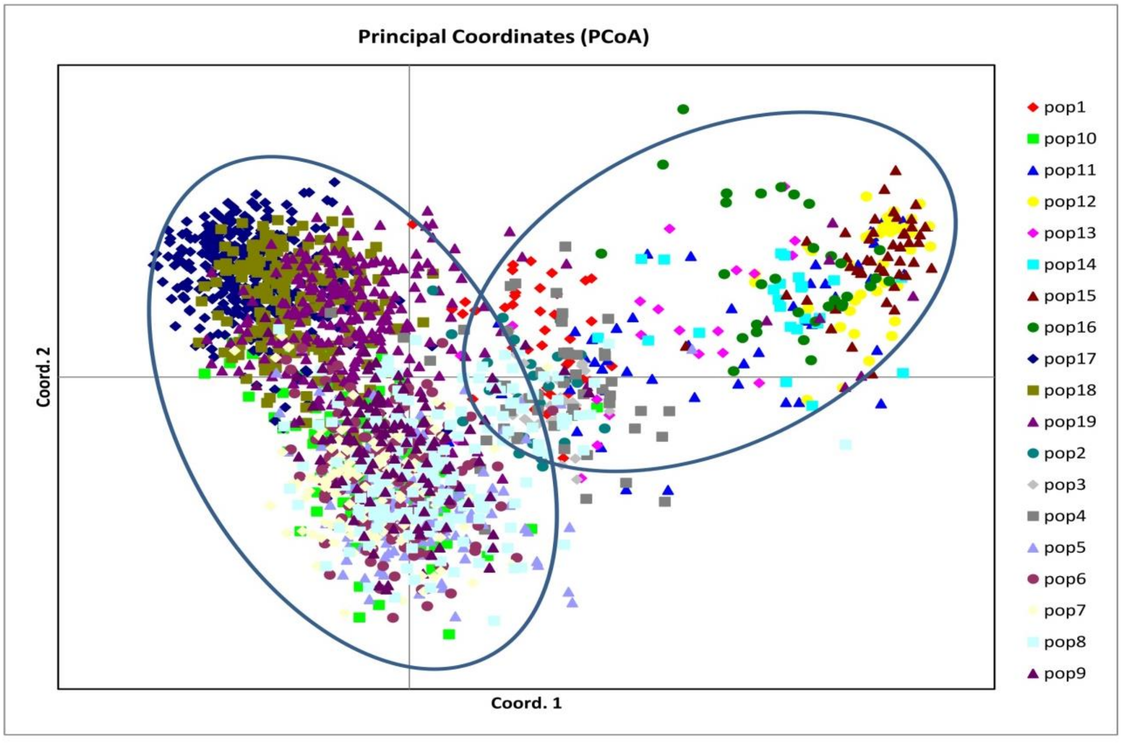

Supplementary Figure S24 PCoA analysis of total east coast rice accessions (2242) (assuming 19 populations)

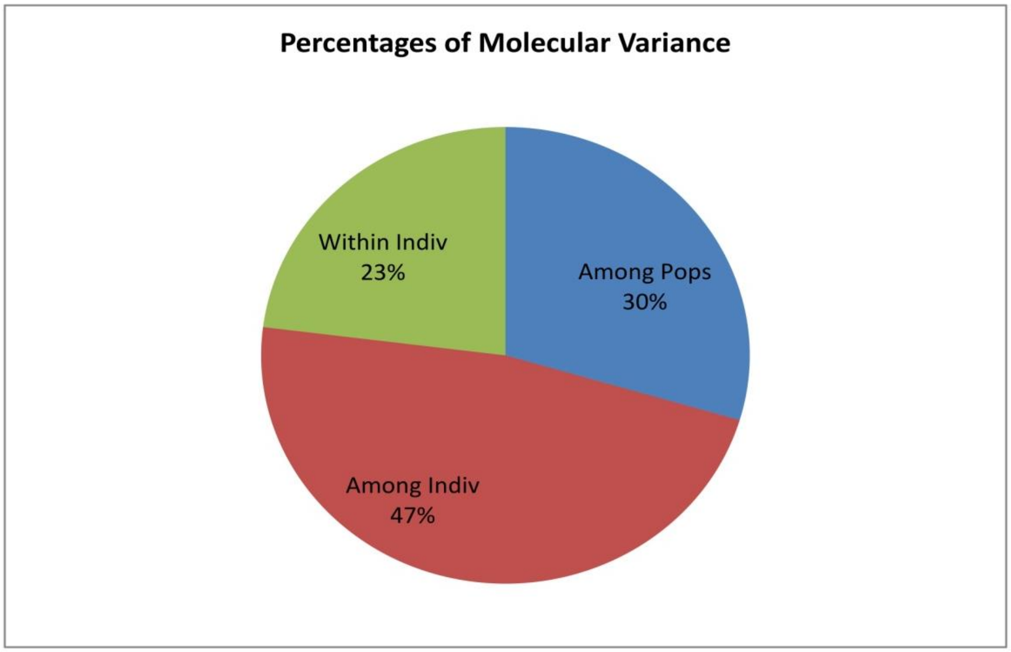

Supplementary figure S25 Pie chart showing percentage of molecular variance of rice collection of Andhra Pradesh

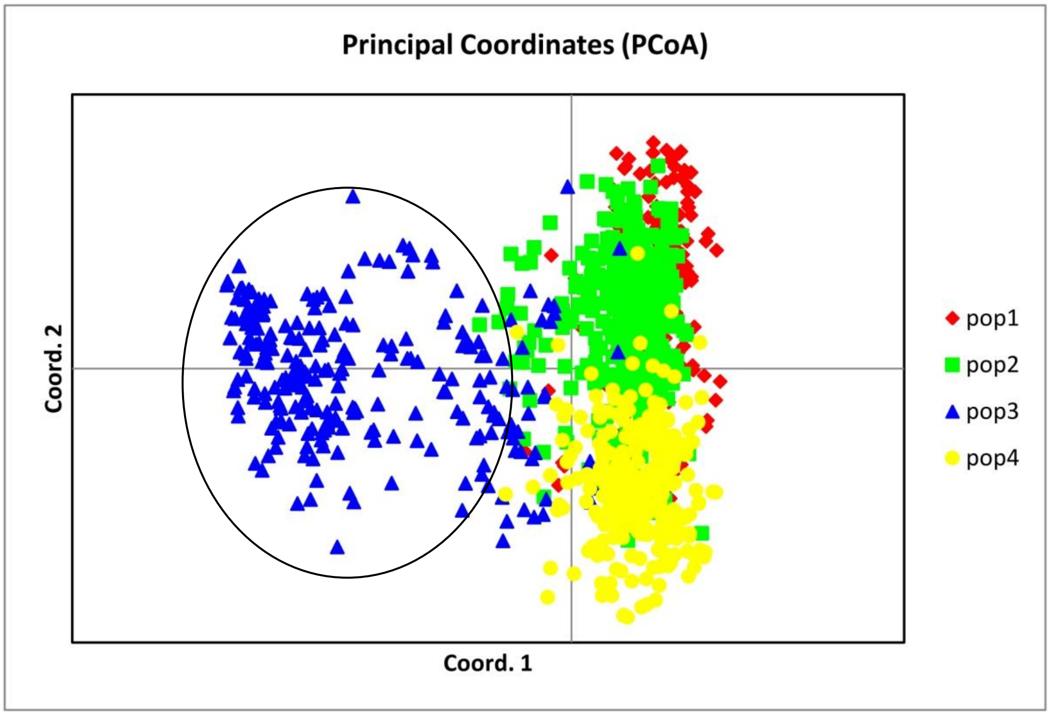

Supplementary figure S26 PCoA analysis of rice collection of Andhra Pradesh

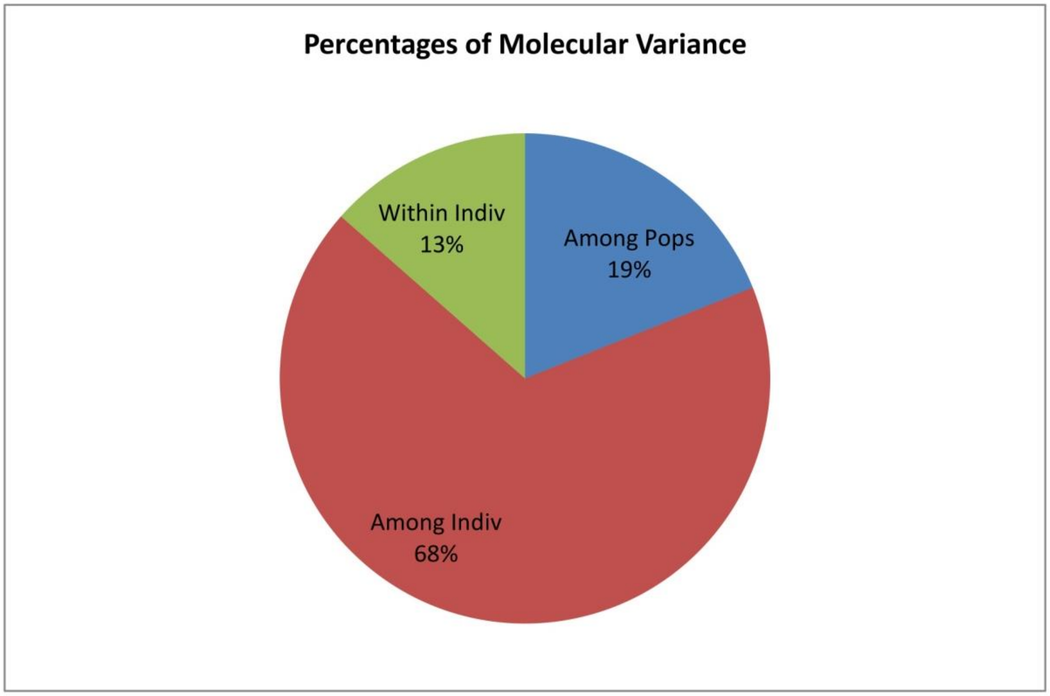

Supplementary figure S27 Pie chart showing percentage of molecular variance of rice collection of Orissa

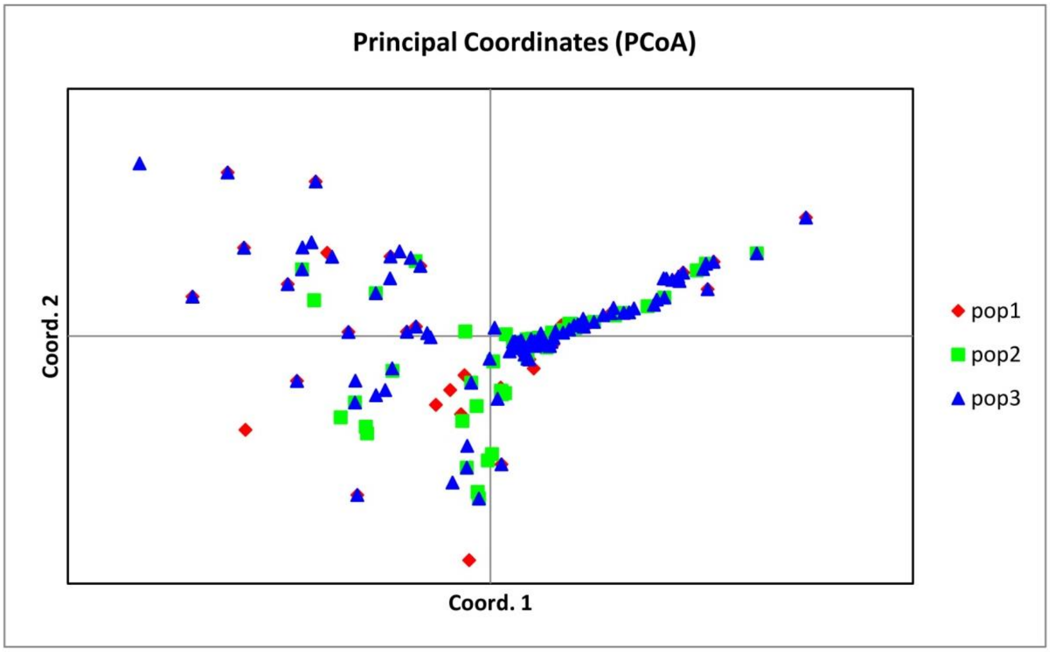

Supplementary figure S28 PCoA analysis of rice collection of Orissa

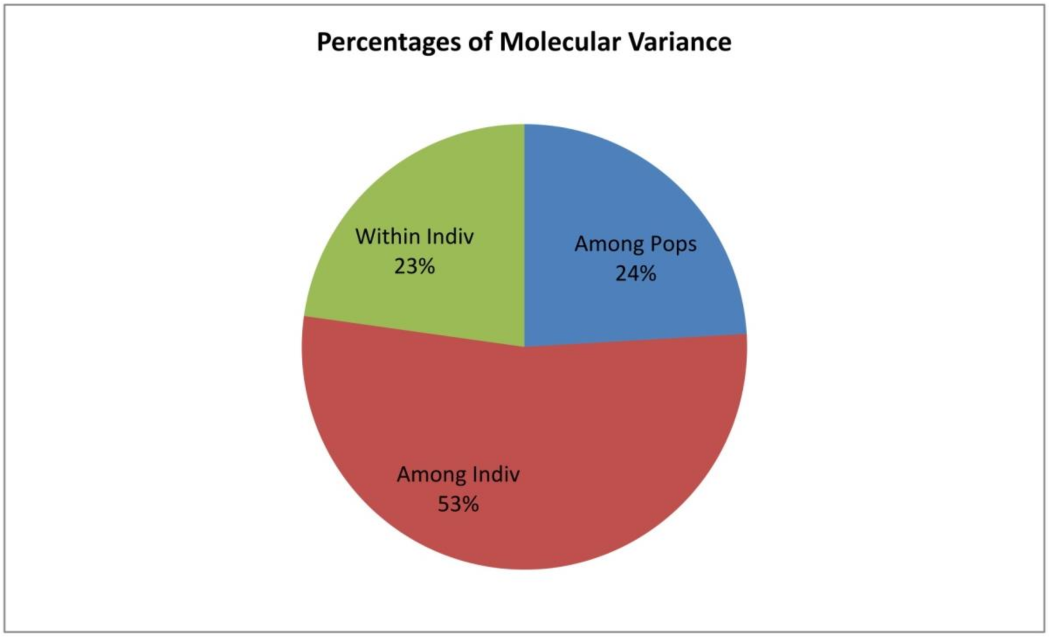

Supplementary figure S29 Pie chart showing percentage of molecular variance of rice collection of Tamil Nadu

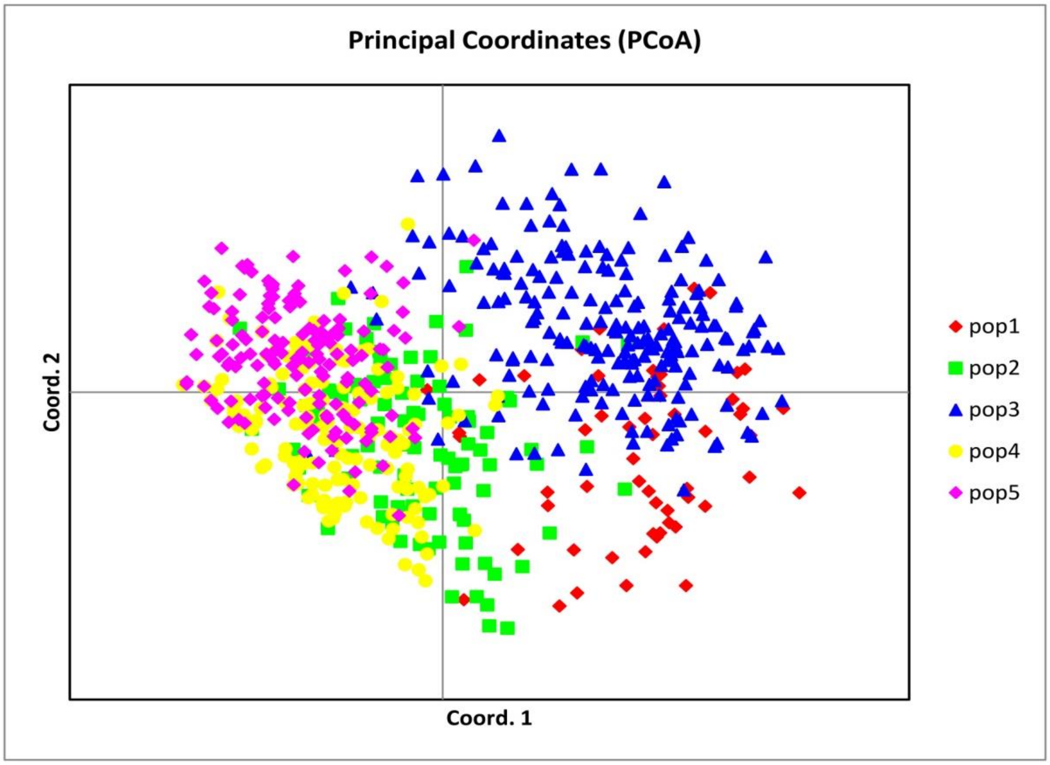

Supplementary figure S30 PCoA analysis of rice collection of Tamil Nadu

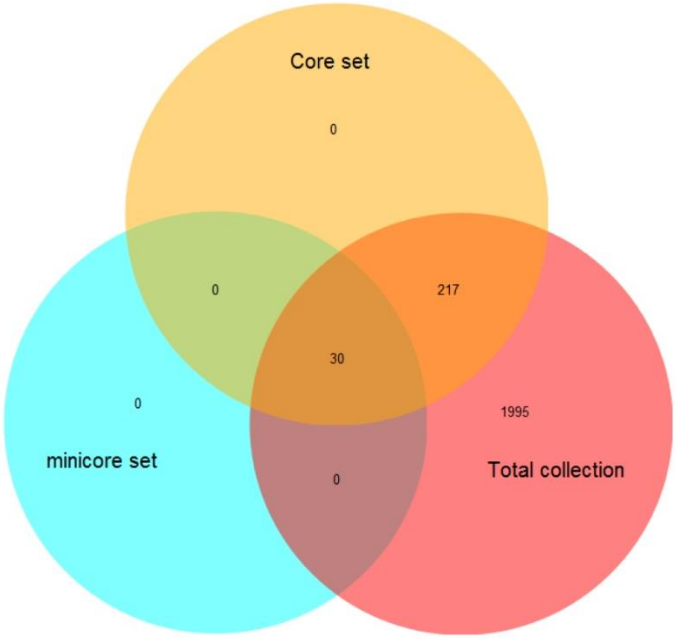

Supplementary Figure S31 Venn diagram showing the distribution of accessions in the east coast core and the mini-core collection

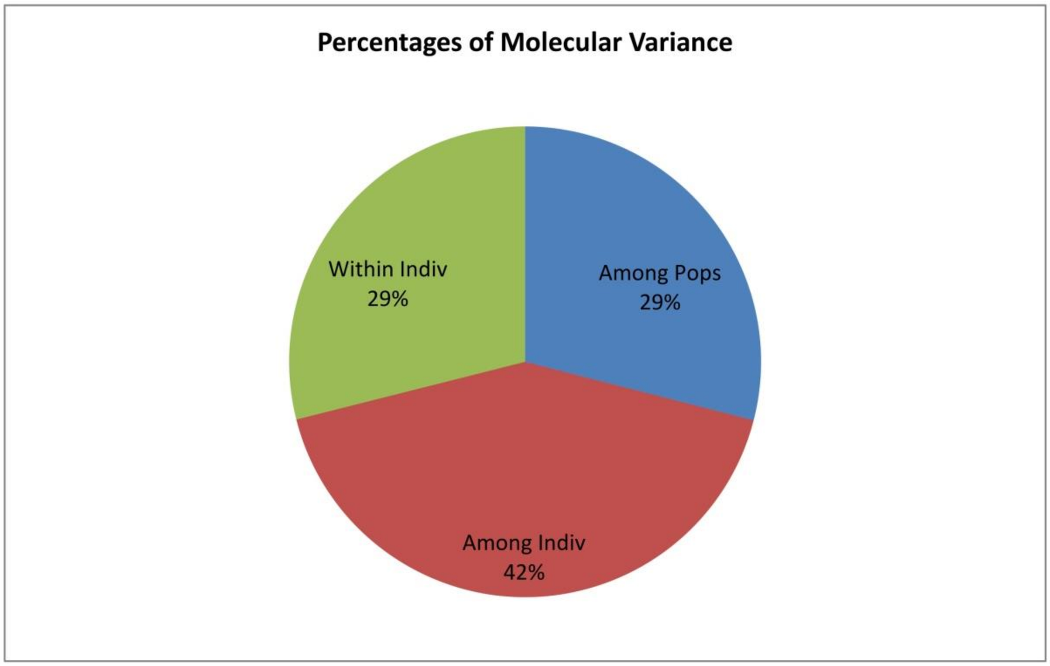

Supplementary Figure S32 Pie chart showing percentage of molecular variance of the east coast rice core set (247 accessions)

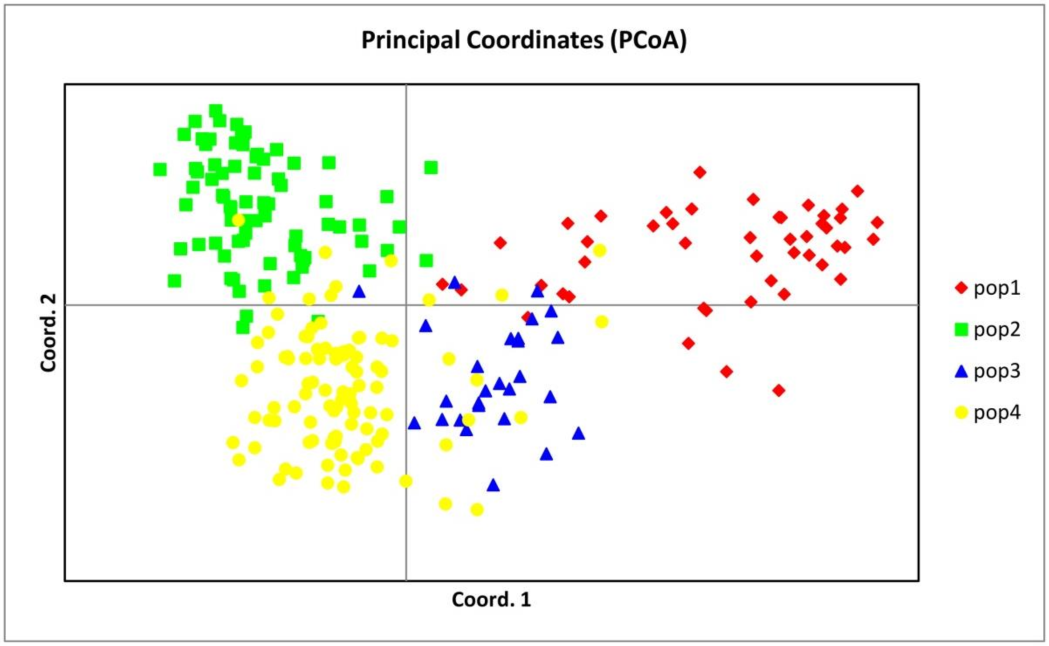

Supplementary Figure S33 PCoA analysis of the east coast rice core set (247 accessions)

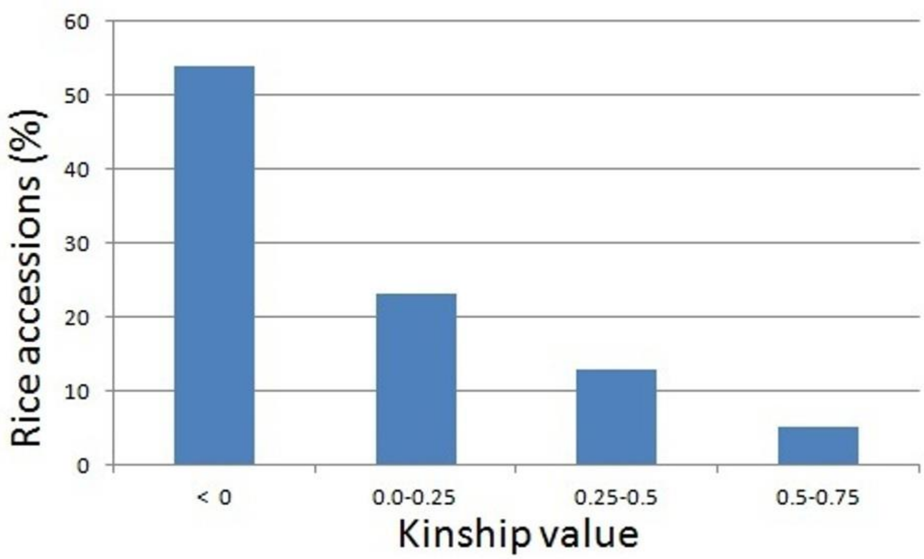

Supplementary Figure S34 Histogram showing the kinship status of rice accessions in the east coast core collection

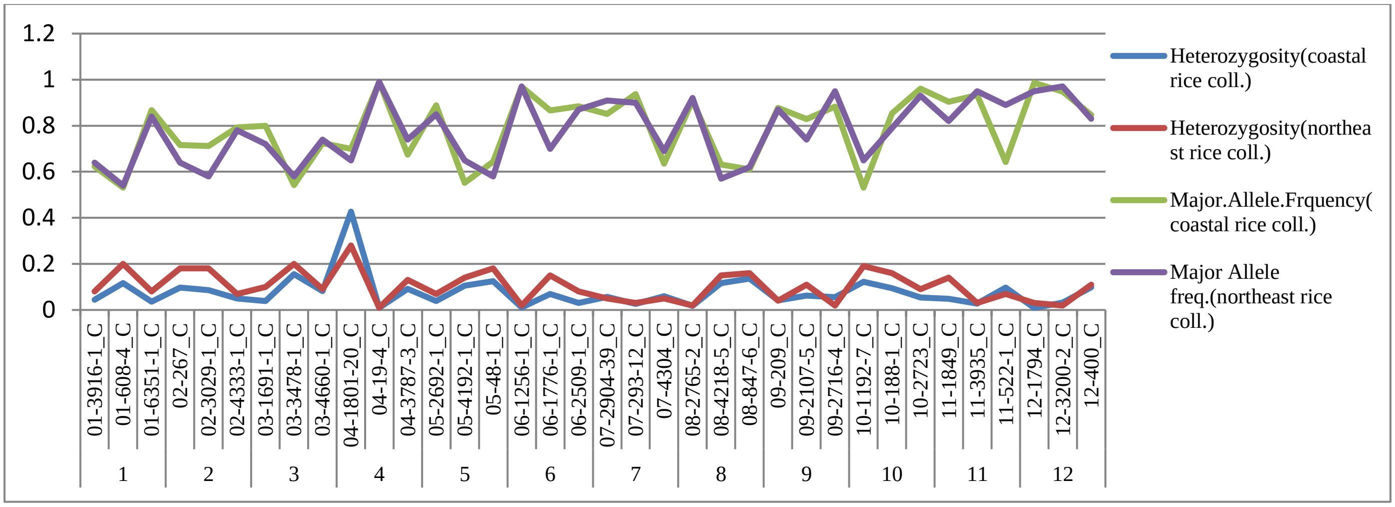

Supplementary Figure S35 Comparative analysis of heterozygosity and major allele frequency values across eat coast rice and north-east rice collection
