# Supplementary material for: Identification of a Diverse Core Set Panel of Rice From the East Coast Region of India Using SNP Markers
Source: Front Genet. 2021 Nov 25;12:726152. doi: 10.3389/fgene.2021.726152 (PMC8655924; doi:10.3389/fgene.2021.726152)
Supplement: Supplementary file 5 [file DataSheet1.docx]

Supplementary Table 4. Mean value of alpha, Fst1, Fst2, Fst3 and Fst4 inferred for the 2242 total east coast collection (model-based approach)

| Mean value of alpha, Fst1, Fst2, Fst3 and Fst4 inferred from model based approach | | | |
| --- | --- | --- | --- |
| Mean value of alpha = 0.0689 |  |  |  |
| Mean value of Fst_1 = 0.5458 |  |  |  |
| Mean value of Fst_2 = 0.2391 |  |  |  |
| Mean value of Fst_3 = 0.4104 |  |  |  |
| Mean value of Fst_4 = 0.3812 |  |  |  |

Supplementary Table 5. Allele-freq. divergence among populations (Net nucleotide distance) for the total east coast collection (computed using point estimates of P)

| Allele-freq. divergence among pops (Net nucleotide distance), computed using point estimates of P. | | |
| --- | --- | --- |
| pop 1 pop2 pop3 pop4 |  |  |
| pop 1 - 0.1226 0.2039 0.1472 |  |  |
| pop 2 0.1226 - 0.2243 0.0540 |  |  |
| pop 3 0.2039 0.2243 - 0.2923 |  |  |
| pop4 0.1472 0.0540 0.2923 - |  |  |

| Supplementary Table 6. The distribution of accessions from three east coast states (Andhra Pradesh, Orissa and Tamil Nadu) assuming the 19 populations | | | | | | | | | | | | | | | | | | | |
| --- | --- | --- | --- | --- | --- | --- | --- | --- | --- | --- | --- | --- | --- | --- | --- | --- | --- | --- | --- |
| **Structure I**  **(4 pop )** | population1 | | | | population2 | | | | | | population3 | | | | | | population4 | | |
| **Total no of accessions** | 179 |  |  |  | 928 |  |  |  |  |  | 235 |  |  |  |  |  | 900 |  |  |
| **Andhra Pradesh** | 39 |  |  |  | 667 |  |  |  |  |  | 219 |  |  |  |  |  | 221 |  |  |
| **Orissa** | 106 |  |  |  | 20 |  |  |  |  |  | 9 |  |  |  |  |  | 242 |  |  |
| **Tamil Nadu** | 34 |  |  |  | 241 |  |  |  |  |  | 7 |  |  |  |  |  | 437 |  |  |
| **Pure** | 128 |  |  |  | 821 |  |  |  |  |  | 157 |  |  |  |  |  | 700 |  |  |
| **admixed** | 51 |  |  |  | 107 |  |  |  |  |  | 78 |  |  |  |  |  | 200 |  |  |
| **Structure II**  **(19 sub populations)** | sub population 1a | sub population1b | sub population1c | sub population 1d | sub population2a | sub population 2b | sub population 2c | sub population 2c | sub population 2e | sub population 2f | sub population3a | sub population 3b | sub population 3c | sub population 3c | sub population 3e | sub population 3f | sub population 4a | sub population 4b | sub population 4c |
| **Total no of accessions** | 49 | 30 | 46 | 54 | 182 | 182 | 158 | 164 | 119 | 122 | 50 | 45 | 30 | 24 | 51 | 35 | 375 | 252 | 273 |
| **Andhra Pradesh** | 9 | 7 | 1 | 23 | 112 | 141 | 133 | 119 | 108 | 50 | 47 | 43 | 28 | 24 | 51 | 26 | 150 | 79 | 67 |
| **Orissa** | 39 | 14 | 45 | 12 | 9 | 1 | 4 | 0 | 1 | 1 | 1 | 0 | 2 | 0 | 0 | 6 | 32 | 53 | 173 |
| **Tamil Nadu** | 1 | 9 | 0 | 19 | 61 | 40 | 21 | 45 | 10 | 71 | 2 | 2 | 0 | 0 | 0 | 3 | 193 | 115 | 33 |
| **pure** | 12 | 10 | 23 | 13 | 27 | 79 | 88 | 78 | 63 | 44 | 2 | 17 | 7 | 11 | 21 | 19 | 150 | 89 | 73 |
| **admixed** | 37 | 20 | 23 | 41 | 155 | 103 | 70 | 86 | 56 | 78 | 48 | 28 | 23 | 13 | 30 | 16 | 225 | 163 | 200 |
|  | cluster 1 | cluster 2 | cluster 3 | cluster 4 | cluster 1 | cluster 2 | cluster 3 | cluster 4 | cluster 5 | cluster 6 | cluster 1 | cluster 2 | cluster 3 | cluster 4 | cluster 5 | cluster 6 | cluster 1 | cluster 2 | cluster 3 |
| **Expected heterozygosity between individuals of the same cluster** | 0.1118 | 0.1583 | 0.0623 | 0.3054 | 0.2756 | 0.2255 | 0.1707 | 0.3797 | 0.202 | 0.2428 | 0.4011 | 0.1735 | 0.3225 | 0.2131 | 0.2164 | 0.2087 | 0.1786 | 0.1826 | 0.3153 |

Supplementary Table 7. Mean value of alpha, Fst1, Fst2, Fst3, Fst4 and Fst5 inferred from model-based approach for the three east coast states (Andhra Pradesh, Orissa and Tamil Nadu) rice collection

| Mean value of alpha, Fst1, Fst2, Fst3, Fst4 and Fst5 inferred from model based approach | | | | | |
| --- | --- | --- | --- | --- | --- |
| Andhra Pradesh | | |  |  |  |
| Mean value of alpha = 0.1075 | | | |  |  |
| Mean value of Fst_1 = 0.4653 | | | |  |  |
| Mean value of Fst_2 = 0.1878 | | | |  |  |
| Mean value of Fst_3 = 0.4460 | | | |  |  |
| Mean value of Fst_4 = 0.3005 | | | |  |  |
|  |  |  |  |  |  |
| Orissa |  |  |  |  |  |
| Mean value of alpha = 0.0468 | | | |  |  |
| Mean value of Fst_1 = 0.0057 | | | |  |  |
| Mean value of Fst_2 = 0.6084 | | | |  |  |
| Mean value of Fst_3 = 0.2258 | | | |  |  |
|  |  |  |  |  |  |
| Tamil Nadu | |  |  |  |  |
| Mean value of alpha = 0.0538 | | | |  |  |
| Mean value of Fst_1 = 0.2077 | | | |  |  |
| Mean value of Fst_2 = 0.2843 | | | |  |  |
| Mean value of Fst_3 = 0.1618 | | | |  |  |
| Mean value of Fst_4 = 0.3611 | | | |  |  |
| Mean value of Fst_5 = 0.3373 | | | |  |  |

Supplementary Table 8. Allele-freq. divergence among pops (Net nucleotide distance) for the three east coast states’ rice collection (computed using point estimates of population)

| Andhra Pradesh |  |  |
| --- | --- | --- |
| pop1 pop2 pop3 pop4 | | |
| pop 1 - 0.0556 0.2852 0.0788 | | |
| pop2 0.0556 - 0.2222 0.0492 | | |
| pop3 0.2852 0.2222 - 0.2519 | | |
| pop4 0.0788 0.0492 0.2519 - | | |
|  | | |

| Orissa |  |  |
| --- | --- | --- |
| Pop1 pop2 pop3 | | |
| pop1 - 0.0997 0.0233 | | |
| pop2 0.0997 - 0.1417 | | |
| pop3 0.0233 0.1417 - | | |

| Tamil Nadu |  |  |  |
| --- | --- | --- | --- |
| pop1 pop2 pop3 pop4 pop5 | | | |
| pop1 - 0.0974 0.0662 0.1044 0.1427 | | | |
| pop 2 0.0974 - 0.0643 0.0331 0.0543 | | | |
| pop3 0.0662 0.0643 - 0.0686 0.0829 | | | |
| pop4 0.1044 0.0331 0.0686 - 0.0244 | | | |
| pop5 0.1427 0.0543 0.0829 0.0244 - | | | |

Supplementary Table 9. Summary of Analysis of molecular variance for 2242 east coast rice collection assuming 4 populations

| Summary AMOVA Table | |  |  |  |  |
| --- | --- | --- | --- | --- | --- |
|  |  |  |  |  |  |
| Source | **df** | **SS** | **MS** | **Est. Var.** | **%** |
| Among Pops | 3 | 5801.603 | 1933.868 | 1.981 | 29% |
| Among Indiv | 2238 | 18656.697 | 8.336 | 3.504 | 51% |
| Within Indiv | 2242 | 2979.500 | 1.329 | 1.329 | 20% |
| Total | 4483 | 27437.800 |  | 6.814 | 100% |

Supplementary Table 10. Principle coordinate analysis showing percentage of variation explained by the first 3 axes of total east coast rice collection

| Percentage of variation explained by the first 3 axes | | |  |
| --- | --- | --- | --- |
|  |  |  |  |
| Axis | **1** | **2** | **3** |
| % | 17.73 | 8.32 | 7.74 |
| Cum % | 17.73 | 26.05 | 33.79 |

Supplementary Table 11. Summary of Analysis of molecular variance for 2242 east coast rice collection assuming 19 populations

| Source | df | | SS | | MS | | Est. Var. | |  |
| --- | --- | --- | --- | --- | --- | --- | --- | --- | --- |
| Among Pops | 18 | 7892.052 | | 438.447 | | 1.898 | | 30% | |
| Among Indiv | 2223 | 16569.988 | | 7.454 | | 3.064 | | 49% | |
| Within Indiv | 2242 | 2971.500 | | 1.325 | | 1.325 | | 21% | |
| Total | 4483 | 27433.540 | |  | | 6.288 | | 100% | |

Supplementary Table 12. Principle coordinate analysis showing percentage of variation explained by the first 3 axes (Assuming 19 sub populations)

| Percentage of variation explained by the first 3 axes | | |  |
| --- | --- | --- | --- |
|  |  |  |  |
| Axis | **1** | **2** | **3** |
| % | 17.73 | 8.30 | 7.73 |
| Cum % | 17.73 | 26.03 | 33.77 |

Supplementary Table 13. Summary of Analysis of molecular variance of the rice collection of east coast states

|  | Summary AMOVA Table | |  |  |  |  |
| --- | --- | --- | --- | --- | --- | --- |
| Andhra Pradesh | Source | **df** | **SS** | **MS** | **Est. Var.** | **%** |
|  | Among Pops | 3 | 3423.265 | 1141.088 | 2.051 | 30% |
|  | Among Indiv | 1129 | 9225.105 | 8.171 | 3.287 | 47% |
|  | Within Indiv | 1133 | 1809.000 | 1.597 | 1.597 | 23% |
|  | Total | 2265 | 14457.370 |  | 6.935 | 100% |
| Orissa | Source | **df** | **SS** | **MS** | **Est. Var.** | **%** |
|  | Among Pops | 2 | 565.222 | 282.611 | 1.162 | 19% |
|  | Among Indiv | 375 | 3416.214 | 9.110 | 4.141 | 68% |
|  | Within Indiv | 378 | 313.000 | 0.828 | 0.828 | 14% |
|  | Total | 755 | 4294.437 |  | 6.131 | 100% |
| Tamil Nadu | Source | **df** | **SS** | **MS** | **Est. Var.** | **%** |
|  | Among Pops | 4 | 1432.129 | 358.032 | 1.240 | 24% |
|  | Among Indiv | 726 | 4830.609 | 6.654 | 2.740 | 53% |
|  | Within Indiv | 731 | 858.500 | 1.174 | 1.174 | 23% |
|  | Total | 1461 | 7121.239 |  | 5.154 | 100% |

Supplementary Table 14. Principal coordinate analyses of rice collection of east coastal states, with percentage of variation explained by the first 3 axes

|  | Percentage of variation explained by the first 3 axes | | |  |
| --- | --- | --- | --- | --- |
|  |  |  |  |  |
| Andhra Pradesh | **Axis** | **1** | **2** | **3** |
|  | **%** | 23.72 | 7.41 | 6.57 |
|  | **Cum %** | 23.72 | 31.14 | 37.70 |
| Orissa | **Axis** | 1 | 2 | 3 |
|  | **%** | 7.52 | 5.99 | 5.55 |
|  | **Cum %** | 7.52 | 13.51 | 19.06 |
| Tamil Nadu | **Axis** | 1 | 2 | 3 |
|  | **%** | 17.78 | 8.79 | 7.02 |
|  | **Cum %** | 17.78 | 26.57 | 33.59 |

Supplementary Table 15. Mean value of alpha, Fst1, Fst2, Fst3 and Fst4 inferred for the 247 east coast core collection (model-based approach)

| Mean value of alpha, Fst1, Fst2, Fst3 and Fst4 inferred from model based approach | | | | |
| --- | --- | --- | --- | --- |
| Mean value of alpha = 0.1035 |  |  |  |  |
| Mean value of Fst_1 = 0.3693 |  |  |  |  |
| Mean value of Fst_2 = 0.3804 |  |  |  |  |
| Mean value of Fst_3 = 0.3772 |  |  |  |  |
| Mean value of Fst_4 = 0.1790 |  |  |  |  |

Supplementary Table 16. Allele-freq. divergence among populations (Net nucleotide distance) of the 247 east coast core collection (computed using point estimates of population)

| Allele-freq. divergence among pops (Net nucleotide distance), computed using point estimates of P. | | | |
| --- | --- | --- | --- |
| 1 2 3 4 |  |  |  |
| 1 - 0.2677 0.1616 0.1945 |  |  |  |
| 2 0.2677 - 0.1466 0.0630 |  |  |  |
| 3 0.1616 0.1466 - 0.1004 |  |  |  |
| 4 0.1945 0.0630 0.1004 |  |  |  |

Supplementary Table 17. Summary of analysis of molecular variance for the 247 east coast core collection (model-based approach)

| Summary AMOVA Table | |  |  |  |  |
| --- | --- | --- | --- | --- | --- |
|  |  |  |  |  |  |
| Source | **df** | **SS** | **MS** | **Est. Var.** | **%** |
| Among Pops | 3 | 768.085 | 256.028 | 2.138 | 29% |
| Among Indiv | 243 | 2025.271 | 8.334 | 3.100 | 42% |
| Within Indiv | 247 | 527.000 | 2.134 | 2.134 | 29% |
| Total | 493 | 3320.356 |  | 7.372 | 100% |

Supplementary Table 18. Percentage of variation explained by the first 3 axes for the 247 east coast core collection (model-based approach)

| Percentage of variation explained by the first 3 axes | | |  |
| --- | --- | --- | --- |
|  |  |  |  |
| Axis | **1** | **2** | **3** |
| % | 19.86 | 8.89 | 6.64 |
| Cum % | 19.86 | 28.74 | 35.38 |
